# A life cycle analysis of the environmental impact of procurement, waste and water in the dental practice

**DOI:** 10.1038/s41415-024-7239-5

**Published:** 2024-04-12

**Authors:** Peter Suresh, John Crotty, Sonja Tesanovic, Othman Alaweed, Sadhbh Doyle, Mikra Kiandee, Emily Hayes, Vanessa Umeh, Bita Khalilinejad, Brett Duane

**Affiliations:** 4141518861001 Undergraduate Student, Dental Science, Dublin Dental University Hospital, Trinity College Dublin, Ireland; 4141518861002 Division of Restorative Dentistry and Periodontology, Dublin Dental University Hospital, Trinity College Dublin, Ireland; 4141518861003 Associate Professor in Dental Public Health, Dublin Dental University Hospital, Trinity College Dublin, Ireland

## Abstract

**Background** Health care is a significant contributor to climate change. Global pressure for a change towards a more sustainable way of providing dental health care has resulted in the creation of the Green Impact Toolkit, which is comprised of a list of suggested changes that dental practices can make to become more sustainable in a number of categories, such as procurement, waste and water.

**Aims** To compare the effectiveness of changes suggested by the Green Impact Toolkit.

**Materials and methods** A comparative life cycle assessment (LCA) was conducted using the Ecoinvent database v3.8 and these data were processed using OpenLCA v1.10.3 software.

**Results** The carbon footprint per patient was significantly reduced after the recommendations were implemented. For instance, using water from a rainwater collection tank instead of the mains supply saved 30 g CO2eq (carbon dioxide equivalents) per patient, a 90% reduction in carbon footprint.

**Discussion **This comparative LCA identified some effective changes which can be easily made by a dental practice. Nevertheless, some actions require some initial financial investment and may be difficult to implement in a busy modern dental practice setting.

**Conclusion **The findings from this study can be used to guide dental practices to making choices which are more sustainable and eco-friendly in the future.

**Supplementary Information:**

Zusatzmaterial online: Zu diesem Beitrag sind unter 10.1038/s41415-024-7239-5 für autorisierte Leser zusätzliche Dateien abrufbar.

## Key points

- The provision of dental health care is a significant contributor to climate change which has led to the search for sustainable solutions which can be implemented in the dental practice.
- This study compares the effectiveness of changes recommended by the Green Impact Toolkit in relation to procurement, waste and water in the dental practice.
- This study provides the dental profession with evidence-based guidance on changes that can be made to reduce their carbon footprint and become more sustainable.

**Supplementary Information:**

Zusatzmaterial online: Zu diesem Beitrag sind unter 10.1038/s41415-024-7239-5 für autorisierte Leser zusätzliche Dateien abrufbar.

## Introduction

Global warming is recognised as one of the greatest challenges faced by our generation. Climate change, an increase in global water and air temperatures, is a constant and insidious process which is resulting in the melting of our ice caps, rising our sea levels at an alarming rate. Furthermore, extreme changes in climate has led to drought, desertification and famine, not to mention the evermore frequent and unprecedented occurrence of natural disasters worldwide.

Greenhouse gas emissions are the main driver of global warming. Efforts are being made by world leaders to tackle this issue. The Intergovernmental Panel on Climate Change highlighted the gravity of the current situation in the *Sixth synthesis report* which urged that deep, rapid and sustained reduction in global greenhouse gas emissions is needed to preclude global climate catastrophe.^1^ The impact of climate change and pollution on health has been extensively explored,^2^ but the impact health care itself has on the environment is often underestimated. Health care's climate footprint makes up 4.4% of net global emissions.^3^ More specifically, in 2014, dentistry in the NHS was found to have a carbon footprint of 675 kilotonnes carbon dioxide equivalents, 3% of its overall carbon footprint.^4^

Many health care organisations, such as the NHS, have committed to reducing their environmental impacts and greenhouse emissions by putting strategies in place to render the delivery of health care more carbon neutral.^5^ Initiatives such as the Sustainable Development Strategy for the Health and Social Care System^6^ will play a pivotal role in reducing the environmental impact of health care on climate change going forward. More importantly, the Green Impact Organisation, also based in the UK, have created a Green Impact Toolkit^7^ to help dental practices make the sustainable choice. This tool is comprised of a list of suggested actions and changes that can be implemented in every dental practice so that they can operate and deliver health care in a more eco-friendly way.

Life cycle assessments (LCAs) offer an evidence-based, standardised method of quantifying the environmental impact of a specific process.^8^ They take into consideration the entire lifetime of a product or activity, from its stage as a raw material, right through to manufacturing, distribution and disposal. It is a useful tool used by researchers worldwide who aim to evaluate the environmental and carbon impact of a product or activity and give a definitive way of comparing and selecting the path of lowest climate impact. This is one of many ways in which we can achieve a carbon neutral delivery of health care in the future.

Prevention of oral diseases must be acknowledged as the most sustainable way for dentistry to progress in the future.^9^ When the carbon footprint of the national dental service in Scotland was analysed, it was found that procurement was found to be the second greatest contributor after travel at 35.9%.^10^ Although energy and waste only contribute to under 16% of the total carbon footprint of dental services,^5^ its contribution must not be overlooked. The aim of this study was to use an LCA to quantitively assess the possible benefits of the actions recommended in the Green Impact Toolkit^7^ under the theme of procurement, waste and water.

This study was carried out in conjunction with and is part of a series of research studies from Dublin Dental University Hospital, which also explore the themes of decontamination, energy and travel. Collectively, these studies will allow dental practices to evaluate which changes are most effective in reducing their carbon footprint at a micro level and will allow us to work together towards a more sustainable way of providing dental health care in the future.

## Materials and methods

An LCA was carried out to quantitively evaluate and compare the impact of actions that can be undertaken in the dental practice to become more sustainable.

### Functional units

A functional unit describes a quantity of a product or product system and the action of this product. They are foundational to LCAs and offer a basis of objective comparison across different products or systems. Seven functional units were selected for this comparative LCA and were taken from the list of recommendations of changes dental practices should make in relation to procurement, waste and water from the Green Impact Toolkit.^7^ This list of recommendations can be found in online Supplementary Appendix 9.

The list of functional units for this comparative LCA can be found in Table 1.

**Table 1:** Functional units

Functional unit		Green Impact Toolkit recommendation
1	Emailing correspondence from the practice associated with one patient	Emailing an appointment notice versus standard practice: posting an appointment notice Emailing a referral letter versus standard practice: posting a referral letter
2	Paper use associated with one patient	Keeping and reusing scrap paper used in patient management versus standard practice: disposing of scrap paper and using new paper Shredding confidential documents used in patient management versus standard practice: shredding all documents Reusing old envelopes used in patient correspondence versus standard practice: buying new envelopes Using paper-only envelopes in patient correspondence versus standard practice: using envelopes with plastic windows Printing and photocopying on double sides of paper used in patient management versus standard practice: printing and photocopying on single sides of two separate sheets of paper
3	Water consumed in toilets in a patient visit	Using dual flush versus standard practice: using regular single flush toilets
4	Water used in the practice during a single patient visit	Using water from a rainwater collection tank versus standard practice: using water from the mains supply
5	Managing the waste from a toothbrush from a patient	Autoclaving, shredding and recycling used toothbrushes versus standard practice: incinerating toothbrush waste
6	Air-water syringes used in a single patient visit	Reusing and sterilising a metal air-water syringe versus standard practice: using a disposable air-water syringe tips at each patient visit
7	Water consumed in washing dishes in staff canteen per patient visit	Using running water versus using a dishwasher versus standard practice: using a filled plugged sink to wash dishes

### System boundaries

System boundaries include the inputs and raw materials, the processes and the outputs and waste products associated with a specific functional unit. This encompasses everything which can contribute to the overall environmental consequence of a product or activity, from the sourcing of the raw materials to the manufacturing and distribution and finally the disposal of waste materials.

The system boundaries of this LCA are illustrated graphically in the form of a flow chart and can be found in online Supplementary Appendix 10.

### Data collection

In order to define the scope of this study, a list of several assumptions were made about the location and size of the dental practice. A list of general assumptions is outlined in online Supplementary Appendix 8.

All weights, except those with external references were weighed with a OHAUS SKX123-EU Scout SKX Portable Balance.^11^

### Functional unit one: emailing correspondence from the practice associated with one patient

#### Green Impact Toolkit recommendation: emailing an appointment notice versus standard practice - posting an appointment notice

It was assumed that one appointment notice was sent to each patient per year.

In the first scenario, the appointment notice was sent by email. It was assumed that the time spent on a computer sending and reading one appointment notice electronically was two minutes. The energy used to access the internet at a speed of 0.2 Mbit/s was included and the email was sent in plain text which has a reduced carbon footprint as opposed to including an attachment file.^12^ The assumed lifespan of the computer was four years.

For inputs and outputs see online Supplementary Appendix 1.1.

Alternatively, the appointment notice was sent by post. This included one sheet of A4 paper which weighed 4.96 g, which was sent in a 100% paper envelope which weighed 4.45 g. The weight of the glue strip and stamp on the envelope was negligible and was not included in the LCA. Transport from the manufacturer of the envelope and the sheet of paper to the dental practice was included in the LCA. The envelope travelled 12.1 km via light freight vehicle to the patient.

For inputs and outputs see online Supplementary Appendix 1.2.

#### Green Impact Toolkit recommendation: emailing a referral letter versus standard practice - posting a referral letter

It was assumed that one referral letter was sent to each patient per year.

In the first scenario, the referral letter was sent by email. It was assumed that the time spent on a computer sending and reading one referral letter electronically is 15 minutes. The energy used to access the internet at a speed of 0.2 Mbit/s was included and the email was sent in plain text which has a reduced carbon footprint as opposed to including an attachment file.^12^ The assumed lifespan of the computer is four years.

For inputs and outputs see online Supplementary Appendix 1.3.

Alternatively, the referral letter was sent by post. This included one sheet of A4 paper which weighed 4.96 g, which was sent in a 100% paper envelope which weighed 4.45 g. The weight of the glue strip and stamp on the envelope was negligible and was not included in the LCA. Transport from the manufacturer of the envelope and the sheet of paper to the dental practice was included in the LCA. The envelope travelled 12.1 km via light freight vehicle to the patient.

For inputs and outputs see online Supplementary Appendix 1.4.

### Functional unit two: paper use associated with one patient

#### Green Impact Toolkit recommendation: keeping scrap paper used in patient management versus standard practice - disposing of scrap paper

Scrap paper was assumed to be any paper which had already been used but was not damaged and could be used again instead of being disposed of. It was assumed that one sheet of A4 scrap paper weighing 4.96 g was kept and reused per patient. Transport from the manufacturer to the practice was included in the LCA. Reusing scrap paper avoided need for disposal.

For inputs and outputs see online Supplementary Appendix 2.1.

Alternatively, all scrap paper was assumed to be treated as non-hazardous waste under Ecoinvent processes.

For inputs and outputs see online Supplementary Appendix 2.2.

#### Green Impact Toolkit recommendation: shredding confidential documents used in patient management versus standard practice - shredding all documents

It was assumed that there was one confidential and one non-confidential A4 sheet of paper weighing 4.96 g associated with each patient.

In the first scenario, a sign was displayed in the practice to instruct staff to shred only confidential documents. A computer was used for 60 seconds to create the sign. The A4 paper used to make the sign weighed 4.96 g and the electricity used to print the paper was included in the LCA.

For inputs and outputs see online Supplementary Appendix 2.3.

One confidential document was shredded and then disposed of in domestic waste. The non-confidential document was not shredded and was disposed of in domestic waste straight away.

For inputs and outputs see online Supplementary Appendix 2.4.

Alternatively, both sheets were shredded and the shredded waste was assumed to be treated as non-hazardous waste under Ecoinvent processes.

For inputs and outputs see online Supplementary Appendix 2.5.

#### Green Impact Toolkit recommendation: re-using old envelopes used in patient correspondence versus standard practice - buying new envelopes

It was assumed that one paper envelope with a plastic window which was made of 4.25 g paper and 0.2 g polyethylene plastic was used for patient correspondence and disposed of in domestic waste after single use.

For inputs and outputs see online Supplementary Appendix 2.6.

Alternatively, the envelope was reused there were two total uses in its lifetime before being disposed and was assumed to be treated as non-hazardous waste under Ecoinvent processes.

For inputs and outputs see online Supplementary Appendix 2.7.

#### Green Impact Toolkit recommendation: using paper-only envelopes in patient correspondence versus standard practice - using envelopes with plastic windows

It was assumed that one 100% paper envelope which weighed 4.45 g was used and disposed of in domestic waste after use.

For inputs and outputs see online Supplementary Appendix 2.8.

Alternatively, a paper envelope with a plastic window was used and was disposed of in domestic waste after use. The envelope was made of 4.25 g of paper and 0.2 g of plastic.

For inputs and outputs see online Supplementary Appendix 2.9.

#### Green Impact Toolkit recommendation: printing and photocopying on double sides of paper used in patient management versus standard practice - printing and photocopying on single sides of two separate sheets of paper

The computer used to create the document and send it to the printer was assumed to work for eight hours a day, five days a week, for 44 weeks per year and has a lifespan of four years and the electricity usage was included in the LCA. The printer used was assumed to have a lifespan of four years and could print 20,000 pages. The electricity and toner usage have been included in the LCA. Printing and photocopying was done on both sides of a single page.

For inputs and outputs see online Supplementary Appendix 2.10.

Alternatively, printing and photocopying was done on single sides of two separate sheets of paper. The sheets of paper were disposed of in domestic waste after use and were incinerated.

For inputs and outputs see online Supplementary Appendix 2.11.

### Functional unit three: water consumed in toilets in a patient visit

#### Green Impact Toolkit recommendation: using dual flush versus standard practice - using regular single flush toilets

It was assumed that each of the five staff members used the toilet three times per day^13^ and 7 out of 15 patients attending the practice used the toilet per day. This was an average of 1.467 flushes per patient.

The dual flush valve weighed 0.52 kg and was manufactured by injection moulding polyvinylchloride plastic.^14^ The valve lasted for ten years before being replaced and disposed of as domestic waste and incinerated.

For inputs and outputs see online Supplementary Appendix 3.1.

The dual flush valve used 3 L of water per flush on the half flush setting.^15^

For inputs and outputs see online Supplementary Appendix 3.2.

The regular flush valve^16^ weighed 0.241 kg and was manufactured by injection moulding polyvinylchloride plastic. The valve lasted for ten years before being replaced and disposed of as domestic waste and incinerated.

For inputs and outputs see online Supplementary Appendix 3.3.

The regular single flush valve used 6 L of water per flush.^17^

For inputs and outputs see online Supplementary Appendix 3.4.

### Functional unit four: water used in the practice during a single patient visit

#### Green Impact Toolkit recommendation: using water from a rainwater collection tank versus standard practice - using water from the mains supply

It was assumed that 36.86 L of water is used per patient per year.^18^ The rainwater harvesting system consisted of a 1,400 L water storage tank made of 43 kg of polyethylene. The water tank was recycled at the end of its 43-year lifespan.

The rainwater was collected by a rainwater collection system consisting of a half open polyvinylchloride plastic gutter^19^ of 32 mm diameter and 30 metres length which was connected to the tank via polyvinylchloride plastic distribution pipes of 32 mm diameter and 6.4 metres length.^20^ Two polyvinylchloride plastic connectors were involved in this system.

A digital luggage scale^21^ was used to measure the weight of the gutter, pipe and connector. A bag was attached to the scale, then the device was turned on so that the weight stayed at 0 g. After that, each item was placed then weighed. The same process was repeated again to ensure the weight was correct.

For inputs and outputs see online Supplementary Appendix 4.1.

A pump was attached to the water tank and used 0.18 kW of energy per minute^22^ to pump 55 L of water to provide supply to the tap for patient use.

For inputs and outputs see online Supplementary Appendix 4.2.

Alternatively, tap water from the mains supply was used. It was assumed that 36.86 L of water is used per patient per year.^18^ The use of normal tap water from the mains supply was included in the LCA.

For inputs and outputs see online Supplementary Appendix 4.3.

### Functional unit five: managing the waste from a toothbrush from a patient per annum

#### Green Impact Toolkit recommendation: autoclaving, shredding and recycling used toothbrushes versus standard practice - incinerating toothbrush waste

It was assumed that the toothbrushes were made of polypropylene plastic. Used toothbrushes were collected at the dental practice from patients. It was assumed that each patient would provide one toothbrush at a visit for disposal.

In the first scenario, the used toothbrushes were recycled. To replicate recycling process, the product was initially transported for shredding and autoclaving. Once decontaminated and following cut off principles, no further burden or benefit of the recycling process was analysed.

For inputs and outputs see online Supplementary Appendix 5.1.

In the second scenario, the used toothbrushes were transported from the dental practice to a specialist waste facility. At this waste facility, brushes were considered contaminated waste and were autoclaved before being shredded.

The toothbrushes were then transported to a separate site of incineration. The transport distance was assumed to be 40 miles between both dental practice and specialist waste facility and between waste facility and incineration plant.^23^

For inputs and outputs see online Supplementary Appendix 5.2.

### Functional unit six: air-water syringes used in a single patient visit

#### Green Impact Toolkit recommendation: re-using and sterilising a metal air-water syringe versus standard practice - using a disposable air-water syringe tips at each patient visit

It was assumed that one metal air-water syringe was used per patient per visit which was made from plastic extrusion and the tip was made from chromium steel. After use, it was sterilised using a washer disinfector and autoclaved. It was assumed one washer disinfector and one autoclave was used per cycle.

For inputs and outputs see online Supplementary Appendix 6.1.

Information on energy use and water use was measured using information from Dublin Dental Hospital sized autoclave and washer disinfectors. The Melag^24^ and Steelco^25^ machines were used to calculate capacity.

For inputs and outputs see online Supplementary Appendix 6.2.

Included in the washer disinfector was 1.32 ml of washer disinfectant (methyl pentane).

For inputs and outputs see online Supplementary Appendix 6.3.

Alternatively, it was assumed that one air-water syringe with disposable plastic tips was used per patient per visit. The air-water syringe tip^26^ was made by injection moulding polypropylene, weighed 6.6 g and was placed in packaging and transported from the manufacturer to the practice and disposed of by incineration after use.

For inputs and outputs see online Supplementary Appendix 6.4.

### Functional unit seven: water consumed in washing dishes in staff canteen per patient visit

#### Green Impact Toolkit recommendation: using running water versus using a dishwasher versus standard practice - using a filled plugged sink to wash dishes

It was assumed that the five staff used one plate, two pieces of cutlery and four mugs each, amounting to 35 items of crockery per day.

In the first scenario, the dishes were manually washed under running tap water using dishwashing soap and a sponge. The amount of water needed to wash 140 items of crockery was 103 L;^27^ therefore, 25.75 L of water was used to wash 35 items.

For inputs and outputs see online Supplementary Appendix 7.1.

A 383 ml bottle of dish soap was used.^28^ The dishwashing soap which included of 30% ethoxylated alcohol, 15% alkylbenzene, 15% isopropanol and 5% octabenzone stabiliser.^29^ Three empty plastic bottles were weighed to get an average of 28.9 g. The bottles were made by injection moulding polyethylene and were disposed of every two months after 600 uses.

For inputs and outputs see online Supplementary Appendix 7.2.

The sponge^30^ was assumed to have been replaced monthly and both were disposed of in domestic waste.

For inputs and outputs see online Supplementary Appendix 7.3.

In the second scenario, the dishes were manually cleaned in a plugged sink using dishwashing soap and a sponge.

A silicone sink plug^31^ was used which was manufactured by injection moulding in Shanghai, China. The LCA included shipping 21,598.024 km from China to Dublin^32^ and light freight vehicle transport to the shop. The plug lasted for ten years before being disposed as domestic waste.

For inputs and outputs see online Supplementary Appendix 7.4.

The energy needed to heat the water was included in the LCA.^33^ The amount of water needed to wash 140 items of crockery was 103 L, therefore 25.75 L of water was used to wash 35 items.^27^ The kitchen sink had a capacity of 15-20 L, the median value of 17.5 L was used;^34^ therefore, two loads were needed to clean and rinse the crockery.

For inputs and outputs see online Supplementary Appendix 7.5.

In the third scenario, the dishwasher was run once a day to wash the 35 pieces of crockery and used one dishwashing tablet. Here, 18.9 L of water and 0.77 kWh of energy was used in the dishwasher per cycle.^33^

The dishwasher was assumed to have lasted for 15 years if run once a day and was disposed of.

For inputs and outputs see online Supplementary Appendix 7.6.

Three dishwasher tablets were weighed to get an average of 20.55 g. The dishwasher tablet contents included ethoxylated alcohol, palm kernel oil, sodium bicarbonate, sodium percarbonate, sodium pyrophosphate and sodium silicate. The LCA included transport of a pack of 30 from Holyhead, Wales to Dublin: 113 km via shipping and 5 km via light freight vehicle and disposal of the plastic polyethylene bag.

For inputs and outputs see online Supplementary Appendix 7.7.

### Data analysis

The LCA was carried out in accordance with the International Organisation of Standardisation guidelines (ISO 14040:2006)^35^ and European Union Product Environmental Footprint 2019 guidance.^36^ The inputs, processes and outputs included in the LCA were taken from the Ecoinvent database v3.8^37^ and these data were processed using OpenLCA v1.10.3 software.^38^

This study examined one impact category which is described in Table 2.

**Table 2:** Impact categories and LCA methods

Impact category	LCA method (units)	Description
Climate change	gCO2eq	Potential for global warming from greenhouse gas emissions

## Results

The results are presented in Table 3. A full breakdown of the contribution analysis of each action can be found in online Supplementary Appendix 11.

**Table 3:** Results (all results given in grams of carbon dioxide equivalents [gCO2eq])

	Green Impact Toolkit recommendation:	Standard practice:	
	**Emailing**	**Posting**	
E-mailing an appointment notice vs posting an appointment notice	0.8	10.6	
	**Emailing**	**Posting**	
E-mailing a referral letter vs posting a referral letter	4.1	22.2	
	**Keeping and reusing scrap paper**	**Disposing scrap paper**	
Keeping and reusing scrap paper used in patient management vs disposing of scrap paper and using new paper	2.8	3.0	
	**Shredding only confidential documents**	Shredding all documents	
Shredding only confidential documents used in patient management vs shredding all documents	3.2	6.3	
	**Re-using old envelopes**	**Buying new envelopes**	
Reusing old envelopes used in patient correspondence vs buying new envelopes	1.9	3.1	
	**Using paper-only envelopes**	**Using envelopes with plastic windows**	
Using paper-only envelopes in patient correspondence vs using envelopes with plastic windows	2.9	3.6	
	**Printing and photocopying on double sides of paper**	**Printing and photocopying on single sides of two separate sheets of paper**	
Printing and photocopying on double sides of paper used in patient management vs printing and photocopying on single sides of two separate sheets of paper	7.0	10.0	
	**Dual flush**	**Regular single flush**	
Using dual flush toilets vs using regular single flush toilets	1.8	4.9	
	**Using water from a rainwater collection**	**Using water from the mains supply**	
Using water from a rainwater collection tank vs using water from the mains supply	3.3	33.3	
	**Autoclaving, shredding and recycling used toothbrushes**	**Incinerating toothbrush waste**	
Autoclaving, shredding and recycling used toothbrushes vs incinerating toothbrush waste	1.9	47.6	
	**Re-using and sterilising a metal air-water syringe tip**	**Using a disposable air-water syringe tip at each patient visit**	
Reusing and sterilising a metal air-water syringe tip vs using a disposable air-water syringe tip at each patient visit	20	58.1	
	**Green Impact Toolkit recommendation**	**Standard practice**	
	**Using running tap water**	**Using a dishwasher**	**Using a filled plugged sink of water**
Using running tap water vs using a dishwasher vs using a filled plugged sink of water to wash dishes in the staff canteen	10.1	76.7	35.7

## Discussion

It was found that there was a marked decrease in carbon footprint across the board when recommendations by the Green Impact Toolkit were implemented. The LCA identified the recommended changes which have the greatest impact when implemented in a practice setting. The effectiveness of each recommendation is presented in Table 4.

**Table 4:** Effectiveness of recommended changes

Green Impact Toolkit recommendation	Amount of carbon saved (gCO2eq)	Percentage decrease in carbon
Emailing an appointment notice instead of posting an appointment notice	9.8	92%
Emailing a referral letter instead of posting a referral letter	18.1	82%
Keeping and reusing scrap paper used in patient management instead of disposing of scrap paper and using new paper	0.2	60%
Shredding only confidential documents used in patient management instead of shredding all documents	3.1	49%
Reusing old envelopes used in patient correspondence instead of buying new envelopes	1.2	39%
Using paper-only envelopes in patient correspondence instead of using envelopes with plastic windows	0.7	19%
Printing and photocopying on double sides of paper used in patient management instead of printing and photocopying on single sides of two separate sheets of paper	3	30%
Using dual flush toilets instead of using regular single flush toilets	3.1	63%
Using water from a rainwater collection tank instead of using water from the mains supply	30	90%
Autoclaving, shredding and recycling used toothbrushes instead of incinerating toothbrush waste	45.7	96%
Reusing and sterilising a metal air-water syringe tip instead of using a disposable air-water syringe tip at each patient visit	38.1	66%
Using running tap water instead of using a dishwasher to wash dishes in the staff canteen	66.6	87%
Using running tap water instead using a filled plugged sink of water to wash dishes in the staff canteen	25.6	72%

The study discovered that emailing appointment notices and referral letters used less carbon when compared to sending appointment and referral letters by physical post, with a 92% and 82% decrease in carbon, respectively. This modern method of communication provides an effective, environmentally conscious solution to patient-related correspondences in the dental practice. However, it must be noted that the benefits must be balanced against the IT security protocols which must be put in place to maintain privacy and this alternative is more susceptible to cyber-attacks and data breaches. Additionally, some patients may not be proficient in the use of computers and may struggle to adjust to modern forms of communication and prefer the traditional way of correspondence with physical letters.

The carbon-saving impact of recommendations associated with reducing paper usage per patient appeared to be relatively lower than other changes a practice can make. However, curbing the paper usage of administrative activities is advantageous from a cost standpoint while simultaneously lessening the practice's environmental impact. It emerged that keeping scrap paper and re-using it was more favourable than printing on new pages from a carbon perspective. Printing on one side of two separate sheets of paper, rather than double-sided printing on a single sheet and choosing to shred all documents rather than just confidential documents used more carbon. Re-using old envelopes as opposed to using a new envelope with each letter expectedly cut down on carbon impacts. Envelopes that have a plastic address window had a greater carbon impact. This can be attributed to the inclusion of plastic in the design; however, this impact on the environment could be offset if the recommendation of reusing envelopes was enforced.

The LCA established that a dual flush toilet used 63% less water and had a lower carbon footprint when compared to a regular flush toilet. This alternative is easy to implement and is a viable way for any practice to make a change to save water and money in the dental practice, while lowering carbon footprint.

It became apparent that collecting rainwater in a tank, rather than using the mains supply, had a markedly lower carbon impact. Despite the high initial investment cost to install the water collecting system, the practice would be compensated in the long run, cutting water usage costs from the main supply while lowering their carbon footprint by 90%. It must be noted that this solution had its limitations. There was no fluoride added to water coming from the tank and it would be difficult to guarantee that the stagnant collected rainwater was free from pathogenic micro-organisms and safe for use and may raise concerns, particularly for patients with a vulnerable immune status.

It was determined that using metal, three-in-one, air-water syringe tips, that were sterilised and reused after each single patient visit, had a lesser carbon impact than using disposable, single-use, air-water syringe tips. Intuitively, single-use, disposable, plastic tips had a much higher carbon footprint; however, the carbon savings were limited by the autoclaving process required to sterilise the metal, air-water syringe tips after each use on a patient.

The hazardous nature of contaminated used toothbrush waste means that they are more difficult to recycle than other items a patient may use. In the UK, and in England in particular, there are guidelines (HTM 07-01)^39^ which provide detailed instructions on the proper handling of medical waste. Contaminated waste must undergo shredding and autoclaving before recycling. The used toothbrushes were considered contaminated waste and therefore must be managed in accordance with these guidelines. The reduction of carbon can be attributed to the polypropylene pellets being recycled after the shredding process, which offset the environmental impact of polypropylene production. This alternative had a 96% lower carbon impact than incinerating the toothbrush waste alone.

Evidence showed that washing dishes by hand in the staff canteen used less carbon than using a dishwasher or a filled sink. This method, however, is more labour intensive* in lieu *of the convenience of using a dishwasher and it may be difficult to achieve staff co-operation. To this end, a rota could be organised to delegate the cleaning duties fairly among the staff, ensuring that they carry out their cleaning responsibilities. Using a filled sink to wash dishes was not the most carbon sparing, but this could be improved if a smaller sink was used which would, in turn, use less water.

The findings from this LCA highlight the cumulative impact the Green Impact Toolkit recommendations can have. However, it must be noted that this study does have its limitations due to the assumptions made and must be used only as guide. It also must be noted that the cornerstone of sustainability in dentistry going forward is prevention of disease and this must not be overlooked. Nevertheless, it can be determined that there are an array of changes that can be made which are easily implementable and cost-effective. Ultimately, if every dental practice were to take on board even some of the actions recommended by the Green Impact Tool, collectively we could to strive to make the deliverance of dental health care more sustainable and we could enter a new age of environmentally friendly dentistry.

## Conclusion

As aforementioned, this study was carried out in conjunction with a series of studies which explored the Green Impact Toolkit suggestions in relation to procurement, waste, water, decontamination, energy and travel. The findings from these studies can be used to guide dental practices to making evidence-based choices which are more sustainable and eco-friendly in the future.

### Supplementary Information

Supplementary Appendices (PDF 3MB)
