# Supplementary material for: A life cycle analysis of the environmental impact of procurement, waste and water in the dental practice
Source: Br Dent J. 2024 Apr 12;236(7):545–51. doi: 10.1038/s41415-024-7239-5 (PMC11014795; doi:10.1038/s41415-024-7239-5)
Supplement: Supplementary file 1 — Supplementary Appendices (PDF 3MB) [file 41415_2024_7239_MOESM1_ESM.pdf]

Supplementary Appendix 1.1

**Inputs and Outputs: Emailed appointment notices**				
**Inputs**				
Flow	Amount	Unit	Provider	Description
operation, computer, laptop, 68% active work with internet access 0.2 Mbit/s	2	min	operation, computer, laptop, 68% active work with internet access 0.2 Mbit/s | operation, computer, laptop, 68% active work with internet access 0.2 Mbit/s | Cutoff, S - RoW	This item represents the computer use to create and send the appointment notice via email.
**Outputs**				
Flow	Amount	Unit	Provider	Description
Emailed appointment notice	1	Item(s)		

Supplementary Appendix 1.2

**Inputs and Outputs: Posted appointment notices**				
**Inputs**				
Flow	Amount	Unit	Provider	Description
kraft paper	0.00496	kg	market for kraft paper | kraft paper | Cutoff, U - RER	This item represents the paper used for one appointment notice Transport from the manufacturer to the office is already included in this item
kraft paper	0.00425	kg	market for kraft paper | kraft paper | Cutoff, U - RER	This item represents one paper envelope used to post the appointment notice Transport from the manufacturer to the office is already included in this item
polyethylene, linear low density, granulate	0.0002	kg	market for polyethylene, linear low density, granulate | polyethylene, linear low density, granulate | Cutoff, U - GLO	This item represents the plastic window on the envelope used to post the appointment notice. Transport from the manufacturer to the office is already included in this item
operation, computer, laptop, 68% active work with internet access 0.2 Mbit/s	1	min	operation, computer, laptop, 68% active work with internet access 0.2 Mbit/s | operation, computer, laptop, 68% active	This item represents the computer use to create and send the appointment notice to the printer. Electricity use is already

			work with internet access 0.2 Mbit/s | Cutoff, S - RoW	included in this item.
printer, laser, black/white	5.00E-05	Item(s)	market for printer, laser, black/white | printer, laser, black/white | Cutoff, U - GLO	This item represents the printer used to print the appointment notice. The electricity and toner usage have already been included in this item.<br>Assumed lifespan 4 years - LCA assumes printer lasts for 20,000 pages -
transport, freight, light commercial vehicle	0.11386	kg*km	transport, freight, light commercial vehicle | transport, freight, light commercial vehicle | Cutoff, U - Europe without Switzerland	The weight of the stamp is negligible so it has not been included in terms of carbon impact.<br>The weight of the paper and total envelope weight is 0.00941kg and the distance travelled is 12.1km.
				0.00941*12.1 = 0.113861
**Outputs**				
Flow	Amount	Unit	Provider	Description
Printed & posted appointment notice	1	Item(s)		
waste graphical paper	0.00496	kg	treatment of waste graphical paper, municipal incineration | waste graphical paper | Cutoff, U - RoW	This item represents the disposal and waste of paper appointment's notice
waste graphical paper	0.00425	kg	treatment of waste graphical paper, municipal incineration | waste graphical paper | Cutoff, U - RoW	This item represents the disposal and waste of the paper portion of the paper envelope
waste polyethylene/polypropylene product	0.0002	kg	treatment of waste polyethylene/polypropylene product, collection for final disposal | waste polyethylene/polypropylene product | Cutoff, U - Europe without Switzerland	This item represents the disposal and waste of the plastic portion of the paper envelope

Supplementary Appendix 1.3

**Inputs and Outputs: Emailed referral letter**				
**Inputs**				
Flow	Amount	Unit	Provider	Description
operation, computer, laptop, 68% active work with internet access 0.2 Mbit/s	15	min	operation, computer, laptop, 68% active work with internet access 0.2 Mbit/s | operation, computer, laptop, 68% active work with internet access 0.2 Mbit/s | Cutoff, U - RoW	This item represents the computer use to create and send the referral letter by email.<br>Electricity use is already included in this item

**Outputs**				
Flow	Amount	Unit	Provider	Description
Email Referral letter	1	Item(s)		

Supplementary Appendix 1.4

**Inputs and Outputs: Posted Referral Letter**				
**Inputs**				
Flow	Amount	Unit	Provider	Description
kraft paper	0.00496	kg	market for kraft paper | kraft paper | Cutoff, U - RER	This item represents the paper used for one referral letter<br>Transport from the manufacturer to the office is already included in this item
kraft paper	0.00425	kg	market for kraft paper | kraft paper | Cutoff, U - RER	This item represents one paper envelope used to post the referral letter<br>Transport from the manufacturer to the office is already included in this item
polyethylene, linear low density, granulate	0.0002	kg	market for polyethylene, linear low density, granulate | polyethylene, linear low density, granulate | Cutoff, U - GLO	This item represents the plastic window on the envelope used to post the referral letter<br>Transport from the manufacturer to the office is already included in this item
operation, computer, laptop, 68% active work with internet access 0.2 Mbit/s	15	min	operation, computer, laptop, 68% active work with internet access 0.2 Mbit/s | operation, computer, laptop, 68% active work with internet access 0.2 Mbit/s | Cutoff, U - RoW	This item represents the computer use to create and send the referral letter to the printer.<br>Electricity use is already included in this item.
printer, laser, black/white	5.00E-05	Item(s)	market for printer, laser, black/white | printer, laser, black/white | Cutoff, U - GLO	This item represents the printer used to print the appointment notice.<br>The electricity and toner usage have already been included in this item.<br>Assumed lifespan 4 years - LCA assumes printer lasts for 20,000 pages
transport, freight, light commercial vehicle	0.11386	kg*km	transport, freight, light commercial vehicle | transport, freight, light commercial vehicle | Cutoff,	The weight of the stamp is negligible so it has not been included in terms of carbon impact.

			U - Europe without Switzerland	The weight of the paper and total envelope weight is 0.00941kg and the distance travelled is 12.1km.
				0.00941*12.1 = 0.113861
**Outputs**				
Flow	Amount	Unit	Provider	Description
Paper Referral letter	1	Item(s)		
waste graphical paper	0.00496	kg	treatment of waste graphical paper, municipal incineration | waste graphical paper | Cutoff, U - RoW	This item represents the disposal and waste of paper referral letter
waste graphical paper	0.00425	kg	treatment of waste graphical paper, municipal incineration | waste graphical paper | Cutoff, U - RoW	This item represents the disposal and waste of the paper portion of the paper envelope
waste polyethylene/poly propylene product	0.0002	kg	treatment of waste polyethylene/polypropylene product, collection for final disposal | waste polyethylene/polypropylene product | Cutoff, U - Europe without Switzerland	This item represents the disposal and waste of the plastic portion of the paper envelope

Supplementary Appendix 2.1

**Inputs and Outputs: Re use scrap paper**				
**Inputs**				
Flow	Amount	Unit	Provider	Description
kraft paper	0.00496	kg	market for kraft paper | kraft paper | Cutoff, U - RER	This item represents one sheet of paper used Transport from the manufacturer to the office is already included in this item
**Outputs**				
Flow	Amount	Unit	Provider	Description
Scrap	1	Item(s)		

Supplementary Appendix 2.2

**Inputs and Outputs: Dispose of scrap paper**				
**Inputs**				
Flow	Amount	Unit	Provider	Description
kraft paper	0.00496	kg	market for kraft paper | kraft paper | Cutoff, U - RER	This item represents one sheet of paper used Transport from the

				manufacturer to the office is already included in this item
**Outputs**				
Flow	Amount	Unit	Provider	Description
Bin Copy	1	Item(s)		
waste graphical paper	0.00496	kg	treatment of waste graphical paper, municipal incineration | waste graphical paper | Cutoff, U - RoW	This item represents the disposal and waste of one sheet of paper

Supplementary Appendix 2.3

**Inputs and Outputs: Paper sign - Shred only confidential documents**				
**Inputs**				
Flow	Amount	Unit	Provider	Description
kraft paper	0.00496	kg	market for kraft paper | kraft paper | Cutoff, U - RER	This item represents the paper used for one sign Transport from the manufacturer to the office is already included in this item
operation, computer, laptop, 68% active work with internet access 0.2 Mbit/s	1	min	operation, computer, laptop, 68% active work with internet access 0.2 Mbit/s | operation, computer, laptop, 68% active work with internet access 0.2 Mbit/s | Cutoff, S - RoW	This item represents the computer use to create and send the sign to the printer. Electricity use is already included in this item Computer price €500 assumed (computer chosen from website)<br>- Assume working 8 hours per day for 5 days per week for 44 weeks per year (allow 8 weeks for AL/bank holidays etc) = total 1760 hours per year<br>- 1760 hours per year X 4 years = 7040 hours per computer lifetime<br>- 7040 hours costs €500; so 500/7040 gives cost for 1 hour = €0.071 per hour to fund the computer<br>Data is for 60 mins of work<br>- €0.071 per hour is the cost for the computer<br>Create and print sign: 60 seconds – divide everything to make it relative to 60 seconds

				- €0.071 divided by 60 = €0.001 – this is the cost of running the computer for 60 seconds<br><br>Cost of computer: €500 https://www.hp.com/ie-en/products/workstations/view-all-workstation-computers.html
printer, laser, black/white	5.00E-05	Item(s)	market for printer, laser, black/white | printer, laser, black/white | Cutoff, U - GLO	This item represents the printer used to print the sign The electricity and toner usage have already been included in this item. Assumed lifespan 4 years - LCA assumes printer lasts for 20,000 pages -
**Outputs**				
Flow	Amount	Unit	Provider	Description
Paper Sign which indicate to shredding	1	Item(s)		

Supplementary Appendix 2.4

**Inputs and Outputs: Disposal of documents**				
**Inputs**				
Flow	Amount	Unit	Provider	Description
kraft paper	0.00496	kg	market for kraft paper | kraft paper | Cutoff, U - RER	This item represents one sheet of paper used Transport from the manufacturer to the office is already included in this item
**Outputs**				
Flow	Amount	Unit	Provider	Description
Bin	1	Item(s)		
waste graphical paper	0.00496	kg	treatment of waste graphical paper, municipal incineration | waste graphical paper | Cutoff, U - RoW	This item represents the disposal and waste of one sheet of paper<br><br>Cost of waste treatment referenced from BD textbook,<br>used domestic waste: 80-123

				pounds per tonne ,<br>median of domestic waste = 101.5 GBP, = exchange rate = 1.15 = 116.725 EUR/ tonne =0.116725 EUR / kg<br><br>Cost for disposing 0.00496kg = 0.000578956 EUR

Supplementary Appendix 2.5

**Inputs and Outputs: Shredding of confidential paper**				
**Inputs**				
Flow	Amount	Unit	Provider	Description
kraft paper	0.00496	kg	market for kraft paper | kraft paper | Cutoff, U - RER	This item represents one sheet of paper used in the shredder<br>Transport from the manufacturer to the office is already included in this item
Paper Sign which indicate to shredding	0.0036	Item(s)		This item represents the sign to indicate the need to shred confidential documents<br><br>Assuming 15 patients per day, working 5 days per week for 44 weeks a year<br>5 multiplied by 44 = 220 working days per year<br>220 multiplied by 15 = 3,300 patients per year<br>3,300 divided by 12 = 275 patients per month<br><br>1 item divided by 275 = 0.0036 of an item - this is the portion of the sign relative to one patient<br>Sign fabrication taken from separate LCA process
Shredding 1 KG waste	0.00496	kg	Shredding 1 KG waste	This item represents the process of shredding one sheet of paper<br>Shredding Obtained from LCA processes<br>Shredding 924kg Uses 36 kWh = €10.8 (36*0.3)
**Outputs**				
Flow	Amount	Unit	Provider	Description

waste graphical paper	0.00496	kg	treatment of waste graphical paper, municipal incineration | waste graphical paper | Cutoff, U - RoW	This item represents the disposal and waste of one sheet of paper

Supplementary Appendix 2.6

**Inputs and Outputs: Shred all documents**				
**Inputs**				
Flow	Amount	Unit	Provider	Description
kraft paper	0.00992	kg	market for kraft paper | kraft paper | Cutoff, U - RER	This item represents two sheets of paper used in the shredder - assuming all documents are being shredded<br>Assuming that two A4 sheets per patient are shredded (confidential and non-confidential<br>Transport from the manufacturer to the office is already included in this item
Shredding 1 KG waste	0.00992	kg	Shredding 1 KG waste	This item represents the process of shredding two sheets of paper<br>Shredding Obtained from LCA processes<br>Shredding 924kg Uses 36 kWh = €10.8 (36*0.3)<br><br>shredding 1kg = 924/10.8 = 0.0116883116883<br>Shredding 0000992kg = 0.00992 * 0.0116883116883 = 0.00011594805 EUR
**Outputs**				
Flow	Amount	Unit	Provider	Description
waste graphical paper	0.00992	kg	treatment of waste graphical paper, municipal incineration | waste graphical paper | Cutoff, U - RoW	This item represents the disposal and waste of two sheets of paper (shredded)<br><br>Cost of waste treatment referenced from BD textbook,<br>used domestic waste: 80-123 pounds per tonne ,<br>median of domestic waste = 101.5 GBP, = exchange rate = 1.15 = 116.725 EUR/ tonne =0.116725 EUR / kg

				Cost for disposing 0.00992kg = 0.001157912 EUR

Supplementary Appendix 2.7

**Inputs and Outputs: Re use second hand envelopes**				
**Inputs**				
Flow	Amount	Unit	Provider	Description
kraft paper	0.00213	kg	market for kraft paper | kraft paper | Cutoff, U - RER	This item represents one paper envelope used Transport from the manufacturer to the office is already included in this item Cost per envelope = €0.04 Cost of paper part only = (4.25/4.45)*0.04<br><br>Everything divided by 2 as this is one envelope which is being re-used, assuming two total uses in the envelope's lifetime Weight of paper part: 0.00425 divided by 2 = 0.002125 Cost of paper part: 0.0019<br><br>Cost of paper envelope: 4c per envelope https://www.vikingdirect.ie/en/packing-mailing-c-107/envelopes-mailing-bags-c-10701
polyethylene, linear low density, granulate	0.0001	kg	market for polyethylene, linear low density, granulate | polyethylene, linear low density, granulate | Cutoff, U - GLO	This item represents the plastic window on the envelope used. Transport from the manufacturer to the office is already included in this item<br><br>Everything divided by 2 as this is one envelope which is being re-used, assuming two total uses in the envelope's lifetime Weight of plastic part: 0.0002 divided by 2 = 0.0001
transport, freight, light commercial vehicle	0.11386	kg*km	transport, freight, light commercial vehicle | transport, freight, light commercial vehicle | Cutoff,	The weight of the stamp is negligible so it has not been included in terms of carbon impact. The weight of the paper and

			U - Europe without Switzerland	total envelope weight is 0.00941kg and the distance travelled is 12.1km.
				0.00941*12.1 = 0.113861
**Outputs**				
Flow	Amount	Unit	Provider	Description
Paper envelope	1	Item(s)		
waste graphical paper	0.00213	kg	treatment of waste graphical paper, municipal incineration | waste graphical paper | Cutoff, U - RoW	This item represents the disposal and waste of the paper portion of the paper envelope<br><br>Everything divided by 2 as this is one envelope which is being re-used, assuming two total uses in the envelope's lifetime
waste polyethylene/polypropylene product	0.0001	kg	treatment of waste polyethylene/polypropylene product, collection for final disposal | waste polyethylene/polypropylene product | Cutoff, U - Europe without Switzerland	This item represents the disposal and waste of the plastic portion of the paper envelope<br><br>Everything divided by 2 as this is one envelope which is being re-used, assuming two total uses in the envelope's lifetime

Supplementary Appendix 2.8

**Inputs and Outputs: Use new envelopes**				
**Inputs**				
Flow	Amount	Unit	Provider	Description
kraft paper	0.00425	kg	market for kraft paper | kraft paper | Cutoff, U - RER	This item represents one paper envelope used<br>Transport from the manufacturer to the office is already included in this item
polyethylene, linear low density, granulate	0.0002	kg	market for polyethylene, linear low density, granulate | polyethylene, linear low density, granulate | Cutoff, U - GLO	This item represents the plastic window on the envelope used.<br>Transport from the manufacturer to the office is already included in this item
transport, freight, light commercial vehicle	0.11386	kg*km	transport, freight, light commercial vehicle | transport, freight, light commercial vehicle | Cutoff, U - Europe without Switzerland	The weight of the stamp is negligible so it has not been included in terms of carbon impact.<br>The weight of the paper and total envelope weight is 0.00941kg and the distance travelled is 12.1km.

				0.00941*12.1 = 0.113861
**Outputs**				
Flow	Amount	Unit	Provider	Description
Paper envelope	1	Item(s)		
waste graphical paper	0.00425	kg	treatment of waste graphical paper, municipal incineration | waste graphical paper | Cutoff, U - RoW	This item represents the disposal and waste of the paper portion of the paper envelope
waste polyethylene/polypropylene product	0.0002	kg	treatment of waste polyethylene/polypropylene product, collection for final disposal | waste polyethylene/polypropylene product | Cutoff, U - Europe without Switzerland	This item represents the disposal and waste of the plastic portion of the paper envelope

Supplementary Appendix 2.9

**Inputs and Outputs: 100% paper envelope**				
**Inputs**				
Flow	Amount	Unit	Provider	Description
kraft paper	0.00445	kg	market for kraft paper | kraft paper | Cutoff, U - RER	This item represents one paper envelope<br>Transport from the manufacturer to the office is already included in this item
transport, freight, light commercial vehicle	0.11386	kg*km	transport, freight, light commercial vehicle | transport, freight, light commercial vehicle | Cutoff, U - Europe without Switzerland	The weight of the stamp is negligible so it has not been included in terms of carbon impact.<br>The weight of the paper and total envelope weight is 0.00941kg and the distance travelled is 12.1km.<br>0.00941*12.1 = 0.113861
**Outputs**				
Flow	Amount	Unit	Provider	Description
Paper envelope	1	Item(s)		
waste graphical paper	0.00445	kg	treatment of waste graphical paper, municipal incineration | waste graphical paper | Cutoff, U - RoW	This item represents the disposal and waste of the paper envelope

**Inputs and Outputs: Plastic window envelopes**				
**Inputs**				
Flow	Amount	Unit	Provider	Description

kraft paper	0.00425	kg	market for kraft paper | kraft paper | Cutoff, U - RER	This item represents one paper envelope used to post the referral letter<br>Transport from the manufacturer to the office is already included in this item
polyethylene, linear low density, granulate	0.0002	kg	market for polyethylene, linear low density, granulate | polyethylene, linear low density, granulate | Cutoff, U - GLO	This item represents the plastic window on the envelope used to post the referral letter<br>Transport from the manufacturer to the office is already included in this item
transport, freight, light commercial vehicle	0.11386	kg*km	transport, freight, light commercial vehicle | transport, freight, light commercial vehicle | Cutoff, U - Europe without Switzerland	The weight of the stamp is negligible so it has not been included in terms of carbon impact.<br>The weight of the paper and total envelope weight is 0.00941kg and the distance travelled is 12.1km.<br>0.00941*12.1 = 0.113861
**Outputs**				
Flow	Amount	Unit	Provider	Description
Paper envelope with plastic window	1	Item(s)		
waste graphical paper	0.00425	kg	treatment of waste graphical paper, municipal incineration | waste graphical paper | Cutoff, U - RoW	This item represents the disposal and waste of the paper portion of the paper envelope
waste polyethylene/polypropylene product	0.0002	kg	treatment of waste polyethylene/polypropylene product, collection for final disposal | waste polyethylene/polypropylene product | Cutoff, U - Europe without Switzerland	This item represents the disposal and waste of the plastic portion of the paper envelope

Supplementary Appendix 2.10

**Inputs and Outputs: Print both sides of one sheet**				
Flow	Amount	Unit	Provider	Description
kraft paper	0.00496	kg	market for kraft paper | kraft paper | Cutoff, U - RER	This item represents the paper used with printing on both sides<br>Transport from the manufacturer to the office is already included in this item
operation, computer, laptop,	1	min	operation, computer, laptop, 68% active work with internet	This item represents the computer use to create and

68% active work with internet access 0.2 Mbit/s			access 0.2 Mbit/s | operation, computer, laptop, 68% active work with internet access 0.2 Mbit/s | Cutoff, S - RoW	send the document to the printer.<br>Electricity use is already included in this item.
printer, laser, black/white	5.00E-05	Item(s)	market for printer, laser, black/white | printer, laser, black/white | Cutoff, U - GLO	This item represents the printer used to print the document.<br>The electricity and toner usage have already been included in this item.<br>Assumed lifespan 4 years - LCA assumes printer lasts for 20,000 pages
**Outputs**				
Flow	Amount	Unit	Provider	Description
Printing both sides	1	Item(s)		
waste graphical paper	0.00496	kg	treatment of waste graphical paper, municipal incineration | waste graphical paper | Cutoff, U - RoW	This item represents the disposal and waste of one sheet of paper

Supplementary Appendix 2.11

**Inputs and Outputs: Print single sides of two sheets**				
**Inputs**				
Flow	Amount	Unit	Provider	Description
kraft paper	0.00992	kg	market for kraft paper | kraft paper | Cutoff, U - RER	This item represents the paper used with printing on one side - all figures have been multiplied by 2 to represent two individual sheets of paper<br>Transport from the manufacturer to the office is already included in this item
operation, computer, laptop, 68% active work with internet access 0.2 Mbit/s	1	min	operation, computer, laptop, 68% active work with internet access 0.2 Mbit/s | operation, computer, laptop, 68% active work with internet access 0.2 Mbit/s | Cutoff, S - RoW	This item represents the computer use to create and send the document to the printer.<br>Electricity use is already included in this item.
printer, laser, black/white	5.00E-05	Item(s)	market for printer, laser, black/white | printer, laser, black/white | Cutoff, U - GLO	This item represents the printer used to print the document.<br>The electricity and toner usage have already been included in this item.<br>Assumed lifespan 4 years - LCA assumes printer lasts for 20,000 pages

**Outputs**				
Flow	Amount	Unit	Provider	Description
Printing one side	1	Item(s)		
waste graphical paper	0.00992	kg	treatment of waste graphical paper, municipal incineration | waste graphical paper | Cutoff, U - RoW	

Supplementary Appendix 3.1

**Inputs and Outputs: Dual flush valve unit**				
**Input**				
Flow	Amount	Unit	Provider	Description
injection moulding	0.52	kg	market for injection moulding | injection moulding | Cutoff, U - GLO	Injection moulding of dual flush valve - Fluidmaster PRO550UK made of complete PVC
polyvinylchloride, suspension polymerised	0.52	kg	polyvinylchloride production, suspension polymerisation | polyvinylchloride, suspension polymerised | Cutoff, U - RER	Plastic used in valve
**Output**				
Flow	Amount	Unit	Provider	Description
Dual Valve Flush Unit Piece	1	Item(s)		
waste polyvinylchloride	0.52	kg	treatment of waste polyvinylchloride, open dump, moist infiltration class (300mm) | waste polyvinylchloride | Cutoff, U - GLO	Regular valve disposal as domestic waste

Supplementary Appendix 3.2

**Inputs and Outputs: Dual flush water usage**				
**Input**				
Flow	Amount	Unit	Provider	Description
tap water	3	kg	tap water production, conventional treatment | tap water | Cutoff, U - Europe without Switzerland	3L is the volume of tap water used for a dual flush toilet on the half flush setting. Assume 1L = 1kg of water therefore mass of tap water used is 3kg. https://www.sawater.com.au/__data/assets/pdf_file/0008/6686/Factsheet_Amenities.pdf

				Tap water costs €0.00187 / L (https://www.water.ie/business/billing/charges) 1 flush = 3L 5 staff members uses toilet 3 times a day (https://www.davidsonmorris.com/toilet-breaks-at-work/). Staff: 15 flushes per day 15 patients per day and every 2nd patient uses toilet (assumption) Patients: total 7 flushes per day (15+7)/15 = 1.467 flushes per patient 3L x 1.467 flushes = 4.401L per patient 4.401L x €0.00187 / L = €0.00823
Dual Valve Flush Unit Piece	1	Item(s)		**Assumption made that valve will last 10 years (2200 days)**
**Output**				
Flow	Amount	Unit	Provider	Description
Dual Flush	1	Item(s)		
wastewater, unpolluted, from residence	3	l	treatment of wastewater, unpolluted, from residence, capacity 1.1E10l/year | wastewater, unpolluted, from residence | Cutoff, U - RoW	3L is the volume of wastewater created for a dual flush toilet on the half flush setting. Assume 1L = 1kg of water therefore mass of wastewater produced is 3kg.

Supplementary Appendix 3.3

**Inputs and Outputs: regular flush valve unit**				
**Input**				
Flow	Amount	Unit	Provider	Description
injection moulding	0.241	kg	market for injection moulding | injection moulding | Cutoff, U - GLO	Injection moulding of regular flush valve - Fluidmaster PRO400UK made of complete PVC in factory
polyvinylchloride, suspension polymerised	0.241	kg	polyvinylchloride production, suspension polymerisation | polyvinylchloride, suspension polymerised | Cutoff, U - RER	Weight of plastic used in valve
**Output**				
Flow	Amount	Unit	Provider	Description

Regular valve flush unit piece	1	Item(s)		
waste polyvinylchloride	0.241	kg	treatment of waste polyvinylchloride, open dump, moist infiltration class (300mm) | waste polyvinylchloride | Cutoff, U - GLO	Regular valve disposal as domestic waste

Supplementary Appendix 3.4

**Inputs and Outputs: Regular flush water usage**				
**Input**				
Flow	Amount	Unit	Provider	Description
tap water	6	kg	tap water production, conventional treatment | tap water | Cutoff, U - Europe without Switzerland	6L is the volume of tap water used for a regular toilet built after 1994 for a single toilet flush (toilet using the regular flush valve). Assume 1L = 1kg of water therefore mass of tap water used is 6kg. https://www.fluidmaster.com/toilet-problems/check-toilets-flush-volume/
Regular valve flush unit piece	1	Item(s)	Regular valve flush unit piece	Cost of the valve ranged from €18.5 (https://www.fixthebog.uk/fluidmaster-pro400uk-bottom-entry-float-valve-fill-valve-1-2-inch-plastic-inlet/?srsltid=AYJSbAegQv_Xb8K9M_PxNFjU74SS6lp9NFDiAB8PDFcAs53pb8AeXGYA3iU) to €33.00 (https://www.sheahans.ie/products/fluidmaster-pro400uk-1-2-inch-bottom-feed-float-valve?variant=36470610100373&currency=EUR&utm_medium=product_sync&utm_source=google&utm_content=sag_organic&utm_campaign=sag_organic&srsltid=AYJSbAeeyfpOiwZvXNoZfaOoMt3MtApnG6Zmh0M9YHJ0GkhlUeDhSgozyYo) – **median is €25.75 per valve** - Assumption made that valve will last 10 years (2200 days)<br>In 10 years practice will see 33000 patients<br>Cost per patient = 25.75/33000 = 0.00078 euros

**Output**				
Flow	Amount	Unit	Provider	Description
Regular Flush	1	Item(s)		
wastewater, from residence	6	l	treatment of wastewater, from residence, capacity 1.1E10l/year | wastewater, from residence | Cutoff, U - RoW	6L is the volume of wastewater created for a regular toilet built after 1994 for a single toilet flush (toilet using the regular flush valve). Assume 1L = 1kg of water therefore mass of wastewater produced is 6kg.

Supplementary Appendix 4.1

**Inputs and Outputs: Making rainwater collection system**				
**Input**				
Flow	Amount	Unit	Provider	Description
injection moulding	43	kg	market for injection moulding | injection moulding | Cutoff, U - GLO	Injection moulding for the water tank. Holds 1400 litre volume
injection moulding	10.46	kg	market for injection moulding | injection moulding | Cutoff, U - GLO	Injection moulding for a gutter made from PVC and a collection pipe Gutter, half open (32mm of diameter * 30 m of length).<br>down pipe (32mm of diameter * 6.4 m of length).
injection moulding	6.8	kg	market for injection moulding | injection moulding | Cutoff, U - GLO	Injection moulding for a Distribution pipe (32 mm of diameter * 23.7 m)
injection moulding	0.05	g	market for injection moulding | injection moulding | Cutoff, U - GLO	Injection moulding for 2 connectors.
polyethylene terephthalate, granulate, bottle grade	43	kg	polyethylene terephthalate, granulate, bottle grade, recycled to generic market for bottle grade PET granulate | polyethylene terephthalate, granulate, bottle grade | Cutoff, U - RoW	Price and weight: https://www.denios.ie/storage-and-dispensing-containers-in-polyethylene-pe-1400-litre-volume-transparent-256911/256911 it is assumed that it is compatible with the connectors and pipes used.
polyvinylchloride, emulsion polymerised	10.46	kg	market for polyvinylchloride, emulsion polymerised | polyvinylchloride, emulsion polymerised | Cutoff, U - GLO	Gutter, half open (32mm of diameter * 30 m of length).<br>down pipe (32mm of diameter * 6.4 m of length).<br>Diameter and length info are taken from the store

polyvinylchloride, emulsion polymerised	6.8	kg	market for polyvinylchloride, emulsion polymerised | polyvinylchloride, emulsion polymerised | Cutoff, U - GLO	Distribution pipe (32 mm of diameter * 23.7 m) Diameter and length info are taken from the store
polyvinylchloride, emulsion polymerised	0.05	g	market for polyvinylchloride, emulsion polymerised | polyvinylchloride, emulsion polymerised | Cutoff, U - GLO	2 connectors added. Assumption: 2 connectors are needed for this water tank system 1 Connector is 32mm in diameter is 0.025 g.
**Output**				
Flow	Amount	Unit	Provider	Description
waste polyethylene terephthalate	43	kg	market for waste polyethylene terephthalate | waste polyethylene terephthalate | Cutoff, U - IE	waste of the water tank
waste polyvinylchloride	17.261	kg	market for waste polyvinylchloride | waste polyvinylchloride | Cutoff, U - IE	waste of the pipes and connectors
Water storage tank system	1	Item(s)		

Supplementary Appendix 4.2

**Inputs and Outputs: Pump in rainwater collection system**				
**Input**				
Flow	Amount	Unit	Provider	Description
water pump operation, electric	0.0019	kWh	market for water pump operation, electric | water pump operation, electric | Cutoff, U - GLO	Power : 0.18 kw (energy that tank pump uses per min to pump 55 L)<br><br>Max capacity (L/min) : 55 L per minute<br><br>36.86/55= 0.670 minutes (amount of water consumed by pt /max water pump delivers per min)<br>0.670/60=0.0111 amount of minutes it takes to pump 36.86 L/60 to convert to amount of hrs needed)<br><br>0.0111* 0.18= 0.0019 kwh (amount of energy consumed per pt)
Water storage tank system	1/(45*220*15)	Item(s)	Water storage tank system	1/(year*week*day*no. pts in a day). Assumption: the tank lasts for 45 yrs. 44 working weeks a year, 5 working

				weeks a day. 15 patients seen a day,
**Output**				
Flow	Amount	Unit	Provider	Description
Water from water storage tank system	36.86	l		Amount of water used per patient

Supplementary Appendix 4.3

**Inputs and Outputs: Mains tap water usage**				
**Input**				
Flow	Amount	Unit	Provider	Description
tap water	36.86	L	tap water production, conventional with biological treatment | tap water | Cutoff, U - Europe without Switzerland	
**Output**				
Flow	Amount	Unit	Provider	Description
Normal tap water usage	1	Item(s)		
wastewater, from residence	36.86	l	treatment of wastewater, from residence, capacity 1.1E10l/year | wastewater, from residence | Cutoff, U - RoW	

Supplementary Appendix 5.1

**Inputs and Outputs: Autoclave, shred and recycle toothbrushes**				
**Input**				
Flow	Amount	Unit	Provider	Description
Autoclave of waste (1kg)	17.87	g	Autoclave of waste (1kg)	Toothbrush considered contaminated waste and is autoclaved at special waste facility. See appendix 6.3
Shredding of waste (1kg)	17.87	g	Shredding of waste (1kg)	Toothbrush is shredded into polypropylene pellets
Transport from use to waste plant (40 miles) one gram	17.87	g	Transport from use to waste plant (40 miles) one gram	Transport of toothbrushes from practice to special waste facility and is recycled , assumed 40miles
**Output**				

Flow	Amount	Unit	Provider	Description

Supplementary Appendix 5.2

**Inputs and Outputs: Incinerate toothbrushes**				
**Input**				
Flow	Amount	Unit	Provider	Description
Autoclave of waste (1kg)	17.87	g	Autoclave of waste (1kg)	Toothbrush considered contaminated waste and is autoclaved at special waste facility. See appendix 6.3.
Shredding of waste (1kg)	17.87	g	Shredding of waste (1kg)	Toothbrush is shredded into polypropylene pellets
Transport from use to waste plant (40 miles) one gram	17.878*2	g	Transport from use to waste plant (40 miles) one gram	Transport of toothbrushes from practice to special waste facility and to incineration plants, assumed 2x40miles
**Output**				
Flow	Amount	Unit	Provider	Description
waste polypropylene	17.87	g	Treatment of waste polypropylene, municipal incineration | waste polypropylene | Cutoff U - RoW	Waste polypropylene pellets are incinerated

Supplementary Appendix 6.1

**Inputs and Outputs: Reusable metal air water syringe**				
**Input**				
Flow	Amount	Unit	Provider	Description
Autoclave resource use per instrument	1	Item(s)	Autoclave resource use per instrument	Autoclave for the sterilisation of the reusable metal 3 in 1 syringe after washing and disinfecting. Assuming 1 autoclave machine needed per cycle. See Appendix 6.2.
extrusion, plastic pipes	(46.94*0.1)/2000	g	extrusion, plastic pipes | extrusion, plastic pipes | Cutoff, U - RER	The manufacturing extrusion process in which the plastic air water syringe is undergoing to be fabricated. For weight assuming we are using the exact amount need for fabrication of plastic disposable syringe.

forging, steel	(46.94*0.9)/2000	g	forging, steel, large open die | forging, steel | Cutoff, U - RoW	Manufacturing process of metal air/water syringe involving the shaping and hammering of the material for the metal under high pressures.
Packaging only including its waste management one instrument	1.0/2000	Item(s)	Packaging only including its waste management one instrument	Packaging used for the product - metal air/water syringe to be shipped and delivered to retailers.
polyethylene terephthalate, granulate, bottle grade	(46.94*0.1)/2000	g	polyethylene terephthalate production, granulate, bottle grade | polyethylene terephthalate, granulate, bottle grade | Cutoff, U - RER	Thermoplastic polymer material used in the production of the plastic packaging.
steel, chromium steel 18/8	(46.94*0.9)/2000	g	steel production, electric, chromium steel 18/8 | steel, chromium steel 18/8 | Cutoff, U - RER	Chromium steel material in metal air/water 3in1 syringe. Utilised in the construction for the metal air/water 3 in 1 syringe tip because of its corrosion resistance, high temperature and tensile strength.
Transport for one instrument per gram	(46.94*0.9)/2000	g	Transport for one instrument per gram	Transport use for shipping and delivery of metal air/water syringe to retail.
Washer disinfector metalclean per one instrument	1	Item(s)	Washer disinfector metalclean	See appendix 6.2
**Output**				
Flow	Amount	Unit	Provider	Description
hazardous waste, for incineration	0.02112+0.00235	g	treatment of hazardous waste, hazardous waste incineration | hazardous waste, for incineration | Cutoff, U - Europe without Switzerland	wastes produced from the use of metal air water syringe tips.<br>Waste treated via incineration
Metal Three in one	1	Item(s)		

Supplementary Appendix 6.2

**Inputs and Outputs: Autoclave and washer disinfector use**				
Flow	Amount	Unit	Provider	Description
Electricity, low voltage	0.03468	Kwh	Market for electricity, low voltage | electricity, low voltage | Cutoff, U - GB	Information average energy usage per cycle: 0.5202 Kwh; 0.5202Kwhr

Electricity, low voltage	0.03072	Kwh	Market for electricity, low voltage | electricity, low voltage | Cutoff, U - GB	Information average energy usage per cycle: Dishwasher1.5054 Kwh
Water, deionised	0.0381	Kg	Market for water, deionised | water, deionised | Cutoff, U - Europe without Switzerland	Information average water usage per cycle: 0.571 Litres
Water, deionised	0.23469	Kg	Market for water, deionised | water, deionised | Cutoff, U - Europe without Switzerland	Information average water usage per cycle: 11.5 Litres
Wastewater, average	0.03810+ 0.23469	L	Market for wastewater, average | wastewater, average | Cutoff, U - Europe without Switzerland	
Methyl Pentane	1.23	ml	Methyl Pentane Washer disinfectant	See appendix 6.3

Supplementary Appendix 6.3

**Methyl Pentane washer disinfectant**				
**Input**				
Flow	Amount	Unit	Provider	
2-methylpentane	0.75	g	Market for 2-methylpentane | 2-methylpentane | Cutoff, U - GLO	
Benzo[thia]diazole-compound	75	g	Market for benzo[thia]diazole-compound | benzo[thia]diazole-compound | Cutoff, U - GLO	
Water, deionised	724	g	Market for water, deionised | water, deionised | Cutoff, U - Europe without Switzerland	
Injection moulding	55	g	Injection moulding | injection moulding | Cutoff, U - RER	
Polypropylene, granulate	55	g	Market for polypropylene, granulate | polypropylene, granulate | Cutoff, U - GLO	
**Output**				
Flow	Amount	Unit	Provider	
Waste polypropylene	55	g	Treatment of waste polypropylene, municipal incineration | waste polypropylene | Cutoff, U - row	

Supplementary Appendix 6.4

**Inputs and Outputs: Disposable air water syringe tips**				
**Input**				

Flow	Amount	Unit	Provider	Description
extrusion, plastic pipes	0.0066	kg	market for extrusion, plastic pipes | extrusion, plastic pipes | Cutoff, U - GLO	The manufacturing extrusion process in which the plastic air water syringe tip is undergoing to be fabricated. For weight assuming we are using the exact amount need for fabrication of plastic disposable syringe.
injection moulding	0.0066	kg		Injection moulding : Plastic Injection moulding is the process of melting plastic pellets.
Packaging only including its waste management one instrument	1	Item(s)	Packaging only including its waste management one instrument	Packaging used to wrap up product of disposable 3 in 1 syringe for shipping to retailer.
polypropylene, granulate	0.0066	Kg	market for polypropylene, granulate | polypropylene, granulate | Cutoff, S - GLO	Thermoplastic polymer material used to fabricate to disposable air/water 3 in1 syringe.
Transport for one instrument per gram	6.6	g	Transport for one instrument per gram	Transport of disposable syringe to retail distance. 6.6g the weight of the product. Distance of transport is 2,139.8km. Transport cost is included into the shipping cost on purchasing.
**Output**				
Flow	Amount	Unit	Provider	Description
Disposable air/water syringe	1	Item(s)		
hazardous waste, for incineration	0.0066	kg	treatment of hazardous waste, hazardous waste incineration | hazardous waste, for incineration | Cutoff, U - Europe without Switzerland	the waste produced from disposal of disposable 3in1 air water syringe tip

Supplementary Appendix 7.1

**Inputs and Outputs: Wash dishes with running water**				
**Input**				
Flow	Amount	Unit	Provider	Description

Dishwashing soap	0.00167	Item(s)	Dishwashing soap	Assumption: change every 2 months: 5 days a week, 8 weeks. = 40 15 patients a day, total last for 600 uses (40x15)
Dishwashing sponge	0.00333	Item(s)	Dishwashing sponge	Assume change every month = 5*4*15 = 300 uses 5 days, 4 weeks, 15 patients daily
heat, air-water heat pump 10kW	0.04007	kWh	heat production, air-water heat pump 10kW | heat, air-water heat pump 10kW | Cutoff, U - Europe without Switzerland	Energy used to heat water
tap water	1.71667	kg	tap water production, conventional with biological treatment | tap water | Cutoff, U - Europe without Switzerland	Amount of tap water used to wash dishes
**Output**				
Flow	Amount	Unit	Provider	Description
Free flow wash	1	Item(s)		
municipal solid waste	0.03553	g	treatment of municipal solid waste, sanitary landfill | municipal solid waste | Cutoff, U - RoW	sponge waste disposed as domestic waste
waste polyethylene/polypropylene product	0.04682	g	treatment of waste polyethylene/polypropylene product, collection for final disposal | waste polyethylene/polypropylene product | Cutoff, U - Europe without Switzerland	Soap bottle Usage of 600 times 5days, 8 weeks, 15 patients daily Soap bottle disposed as Domestic waste
wastewater, from residence	1.71667	l	treatment of wastewater, from residence, capacity 1.1E10l/year | wastewater, from residence | Cutoff, U - RoW	Amount of tap water used to wash dishes

Supplementary Appendix 7.2

**Inputs and Outputs: Dishwashing soap bottle and contents**				
**Input**				
Flow	Amount	Unit	Provider	Description
alkylbenzene, linear	66.339	g	alkylbenzene production, linear | alkylbenzene, linear | Cutoff, U - RER	15% of soap
ethoxylated alcohol (AE11)	132.678	g	ethoxylated alcohol (AE11) production, palm oil |	30% of soap

			ethoxylated alcohol (AE11) | Cutoff, U - RER	
injection moulding	28.09	g	injection moulding | injection moulding | Cutoff, U - RER	
isopropanol	66.339	g	isopropanol production | isopropanol | Cutoff, U - RER	15% of soap
octabenzone	22.113	g	market for octabenzone | octabenzone | Cutoff, U - GLO	Stabiliser - undisclosed weight assume 5%
polyethylene, low density, granulate	28.09	g	polyethylene production, low density, granulate | polyethylene, low density, granulate | Cutoff, U - RER	Average of 3 empty bottles of 500ml 28.00, 27.27, 29.00
**Output**				
Flow	Amount	Unit	Provider	Description
2-Octanone	22.113	g		
Alcohols, c12-14, ethoxylated	132.678	g		
Alkylbenzene (c10-c15)	66.339	g		
Dishwashing soap	1	Item(s)		
Isopropalin	66.339	g		
waste polyethylene/polypropylene product	28.09	g	treatment of waste polyethylene/polypropylene product, collection for final disposal | waste polyethylene/polypropylene product | Cutoff, U - Europe without Switzerland	plastic bottle waste

Supplementary Appendix 7.3

**Inputs and Outputs: Sponge**				
**Input**				
Flow	Amount	Unit	Provider	Description
orthophthalic acid based unsaturated polyester resin	0.006	kg	orthophthalic acid based unsaturated polyester resin production | orthophthalic acid based unsaturated polyester resin | Cutoff, U - RER	Soft part of sponge
polyurethane, flexible foam, flame retardant	0.00466	kg	polyurethane production, flexible foam, TDI-based, flame retardant | polyurethane, flexible foam, flame retardant | Cutoff, U - RoW	Hard scrubber of sponge
transport, freight, light commercial vehicle	285.0 * 0.0106	kg*km	transport, freight, light commercial vehicle | transport, freight, light commercial vehicle | Cutoff, U - Europe without Switzerland	

**Output**				
Flow	Amount	Unit	Provider	Description
Dishwashing sponge	1	Item(s)		

Supplementary Appendix 7.4

**Inputs and Outputs: Sink plugger**				
**Input**				
Flow	Amount	Unit	Provider	Description
injection moulding	0.02773	kg	injection moulding | injection moulding | Cutoff, U - RER	Manufacturing silicone plug process
silicone product	0.02773	kg	market for silicone product | silicone product | Cutoff, S - RER	Sink plug material
transport, freight, light commercial vehicle, EURO1	0.15252	kg*km	transport, freight, light commercial vehicle, EURO1 | transport, freight, light commercial vehicle, EURO1 | Cutoff, U - ZA	Transport Dublin port to Jervis
transport, freight, sea, container ship with reefer, cooling	598.913	kg*km	transport, freight, sea, container ship with reefer, cooling | transport, freight, sea, container ship with reefer, cooling | Cutoff, U - GLO	Transport from shanghai port to Dublin port = 11662 nautical miles =21598.024 km
**Output**				
Flow	Amount	Unit	Provider	Description
Silicone sink plug	1	Item(s)		-

Supplementary Appendix 7.5

**Inputs and Outputs: Wash dishes with plugged sink**				
**Input**				
Flow	Amount	Unit	Provider	Description
Dishwashing soap	0.00167	Item(s)	Dishwashing soap	Assumption: change every 2 months: 5 days a week, 8 weeks. = 40 15 patients a day, total last for 600 uses (40x15)
Dishwashing sponge	0.00333	Item(s)	Dishwashing sponge	Assume change every month = 5*4*15 = 300 uses 5 days, 4 weeks, 15 patients daily
heat, air-water heat pump 10kW	0.05447	kWh	heat production, air-water heat pump 10kW | heat, air-water heat pump 10kW |	Energy used to heat water Electricity usage per patient = 0.023342984933333*2.333333333 = 0.054466964 kWh

			Cutoff, U - Europe without Switzerland	
Silicone sink plug	3.03E-05	Item(s)	Silicone sink plug	Assume last 10 years. 5*44*10*15 = 33000 5days 44 working weeks 10 years 15 patients https://brightrubberplastic.en.made-in-china.com/product/SOPfHihbfBkR/China-Rubber-Sink-Plug-Metal-Handle-Silicone-Stoppers-Rubber-Drain-Plug.html Cost of sink plug = 0.5 EUR Cost of sink plug per patient = 0.5/33000 = 0.0000151515 EUR
tap water	2.33333	kg	tap water production, conventional with biological treatment | tap water | Cutoff, U - Europe without Switzerland	A kitchen sink holds about 15 – 20 litres of water in them. so we use the median of 17.5 l . , to wash 35 items, requires 25.75L therefore needs 2 fills. = 17.5*2 divided by 15 because 15 patients seen daily
**Output**				
Flow	Amount	Unit	Provider	Description
Sink plug wash	1	Item(s)		
municipal solid waste	0.533	g	treatment of municipal solid waste, sanitary landfill | municipal solid waste | Cutoff, U - RoW	sponge waste disposed as domestic waste
municipal solid waste	0.02773	kg	treatment of municipal solid waste, sanitary landfill | municipal solid waste | Cutoff, U - RoW	Silicone plug waste disposed as domestic waste
waste polyethylene/polypropylene product	0.70225	g	treatment of waste polyethylene/polypropylene product, collection for final disposal | waste polyethylene/polypropylene product | Cutoff, U - Europe without Switzerland	Soap bottle Usage of 600 times 5days, 8 weeks, 15 patients daily Soap bottle disposed as Domestic waste
wastewater, from residence	2.33333	l	treatment of wastewater, from residence, capacity 1.1E10l/year | wastewater, from residence | Cutoff, U - RoW	

Supplementary Appendix 7.6

**Inputs and Outputs: Wash dishes in dishwasher**				

**Input**				
Flow	Amount	Unit	Provider	Description
dishwasher	2.02E-05	Item(s)	market for dishwasher | dishwasher | Cutoff, U - GLO	lifespan = 15years Used for: 5(days)x 44 (weeks) x15(years) = 3600 days already includes disposal in LCA process
Dishwashing tablet	0.06667	Item(s)	Dishwashing tablet	Weighed 3 tablets ; 21.98, 18.00, 20.55 Avg = 20.18
electricity, low voltage	0.05133	kWh	market for electricity, low voltage | electricity, low voltage | Cutoff, U - IE	Energy used to run dishwasher 0.77kWh Electricity usage per patient = 0.77/15 = 0.05133 kWh
tap water	1.26	kg	tap water production, conventional with biological treatment | tap water | Cutoff, U - Europe without Switzerland	amount of water used in a dishwasher (18.9l) per patient = 18.9/15 = 1.26l
**Output**				
Flow	Amount	Unit	Provider	Description
Dishwashing cutlery	1	Item(s)		
wastewater, from residence	1.26	l	treatment of wastewater, from residence, capacity 1.1E10l/year | wastewater, from residence | Cutoff, U - RoW	

Supplementary Appendix 7.7

**Inputs and Outputs: Dishwasher tablet**				
**Input**				
Flow	Amount	Unit	Provider	Description
ethoxylated alcohol (AE11)	1.009	g	ethoxylated alcohol (AE11) production, palm oil | ethoxylated alcohol (AE11) | Cutoff, U - RER	
injection moulding	0.41933	g	injection moulding | injection moulding | Cutoff, U - RER	30 tablets per bag
palm kernel oil, crude	1.009	g	palm oil mill operation | palm kernel oil, crude | Cutoff, U - RoW	
polyethylene, low density, granulate	0.41933	g	polyethylene production, low density, granulate | polyethylene, low density, granulate | Cutoff, U - RER	12.58g empty bag. Full packet, 30 tablets in each packet 1 tablet uses plastic of 12.58/30
sodium bicarbonate	10.09	g	soda production, solvay process | sodium bicarbonate | Cutoff, U - RER	

sodium percarbonate, powder	4.036	g	market for sodium percarbonate, powder | sodium percarbonate, powder | Cutoff, U - RER	
sodium pyrophosphate	1.009	g	sodium pyrophosphate production | sodium pyrophosphate | Cutoff, U - GLO	
sodium silicate, spray powder, 80%	1.009	g	sodium silicate production, spray powder, 80% | sodium silicate, spray powder, 80% | Cutoff, U - RER	
transport, freight, light commercial vehicle, EURO1	0.11099	kg*km	transport, freight, light commercial vehicle, EURO1 | transport, freight, light commercial vehicle, EURO1 | Cutoff, U - ZA	Transport Dublin port to Jervis avg = 20.18g weight of tablets Distance = 5.5km
transport, freight, sea, container ship with reefer, cooling	2.28034	kg*km	transport, freight, sea, container ship with reefer, cooling | transport, freight, sea, container ship with reefer, cooling | Cutoff, U - GLO	Transport Holyhead to Dublin port (ship) = 113km Avg weight of tablet - 20.18g
**Output**				
Flow	Amount	Unit	Provider	Description
Alcohols, c12-14, ethoxylated	1.009	g		
Bicarbonate, ion	10.09	g		
Detergents, oil	1.009	g		palm oil waste
Dishwashing tablet	1	Item(s)		
Hydrogen peroxide	1.3117	g		Sodium percarbonate contains 32.5% of Hydrogen peroxide by weight 4.036 * 0.325 = 1.3117g
Pyrophosphate	1.009	g		
Silicate particles	1.009	g		
sodium carbonate	2.7243	g		Sodium percarbonate contains 67.5% Sodium Carbonate 4.036 * 0.675 = 2.7243g
waste polyethylene/poly propylene product	0.41933	kg	treatment of waste polyethylene/polypropylene product, collection for final disposal | waste polyethylene/polypropylene product | Cutoff, U - Europe without Switzerland	Waste product of plastic packaging per tablet

Supplementary Appendix 8

List of General Assumptions

Item	Assumption

**Setting and location of dental practice**	• Fictional General dental practice<br>• Located at Lincoln Place, Dublin 2, Ireland<br>• 15 patients per day<br>• 5 staff members: 2 dentists, 2 nurses, 1 receptionist<br>• Practice whole building size is 100sq m<br>• Each individual dental surgery is 10sq m<br>• Assume working 8 hours per day for 5 days per week for 44 weeks per year (allow 8 weeks for AL/bank holidays etc) = total 1760 hours per year
**Size of the practice**	• Practice whole building size is 100sq m<br>• Each individual dental surgery is 10sq m<br>• 15 patients a day<br>• 44 working weeks a year<br>• Working 5 days a week
**Travel assumptions**	• Patients travel 7.52miles one way - 7.52 miles = 12.1km<br>• Staff travel 21 miles one way

Supplementary Appendix 9

Green Impact Toolkit Theme: Procurement, waste and water

Procurement, waste and water	EITHER the practice does not have high water pressure<br>OR<br>it has fitted [water saving valves] or requested that they be fitted to reduce water pressure.
Procurement, waste and water	If the practice has a kitchen or access to a shared kitchen it has taken action to ensure at least two of the following:<br>• The dishwasher is only run when full;<br>• Crockery is not washed under a running tap;<br>• All sinks have working plugs;<br>• All sinks have washing up bowls.
Procurement, waste and water	Any dual-flushing toilets have been labelled so that users know which button is for the half flush.
Procurement, waste and water	Consider water storage solution

Procurement, waste and water	The practice is reducing the number of letters sent out by, or on behalf of, the practice in envelopes that contain plastic windows and are avoiding using poly-wrap alternatives.
Procurement, waste and water	EITHER the practice does not have any bottle-fed water coolers OR if it does, it has a valid reason for not having replaced bottle-fed water coolers with mains-fed versions, or removed them completely.
Procurement, waste and water	The practice actively participates in the [https://www.terracycle.co.uk/en-GB/brigades/colgate/brigade_faqs|Colgate Terracycle Scheme] and encourages its staff and patients to use it.
Procurement, waste and water	SMS/email is used to contact patients where possible, rather than paper mail (subject to patient agreement).
Procurement, waste and water	All staff are encouraged to print and photocopy on both sides of paper. Printers and photocopiers have been set to [duplex] as default wherever possible.

Procurement, waste and water	Internal documents are scanned and emailed for distribution, where appropriate, rather than printed and mailed to reduce printing.
Procurement, waste and water	Any single-sided printed paper not containing patient details is used for scrap paper within the practice.
Procurement, waste and water	There are signs above confidential waste containers and/or shredders questioning staff about whether the item really needs to be shredded and all staff have been reminded of what constitutes confidential waste.
Procurement, waste and water	The practice collects incoming envelopes and reuses them in preference to buying new envelopes.
Procurement, waste and water	The practice is actively taking measures to conserve water.
Procurement, waste and water	Practices actively separate clinical sterile wrappers (paper and plastic parts) and recycle these.

Procurement, waste and water	The dental team should not consider single-use goods unless mandated through patient safety and/or legislation.
Procurement, waste and water	The practice has completed calculations to estimate the impact of their patients taking part in the [https://www.terracycle.co.uk/en-GB/brigades/colgate/brigade_faqs|Colgate Terracycle Scheme] (see B023).

Supplementary Appendix 10

Comprehensive list of system boundary diagrams for all functional units.

FIGURE 1: System Boundary Diagram for email referral letter

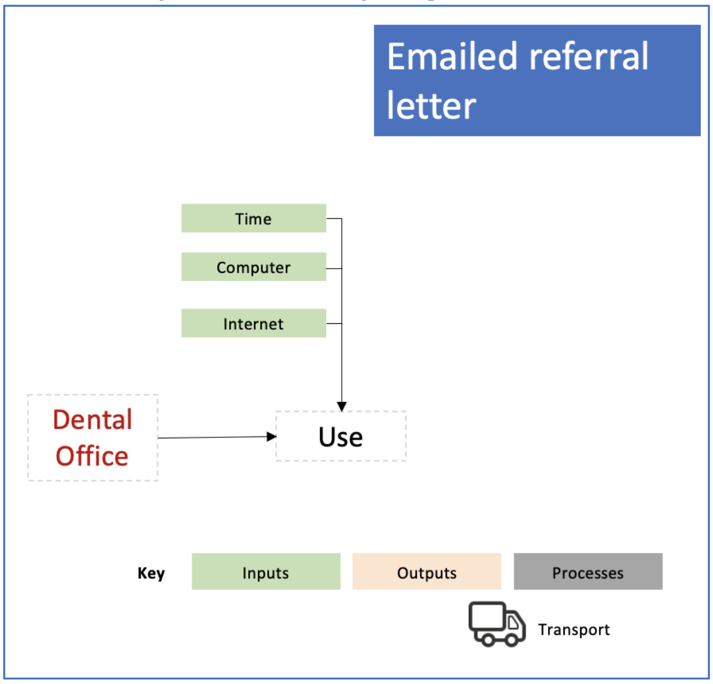

FIGURE 2: System Boundary Diagram for printed and posted referral letter

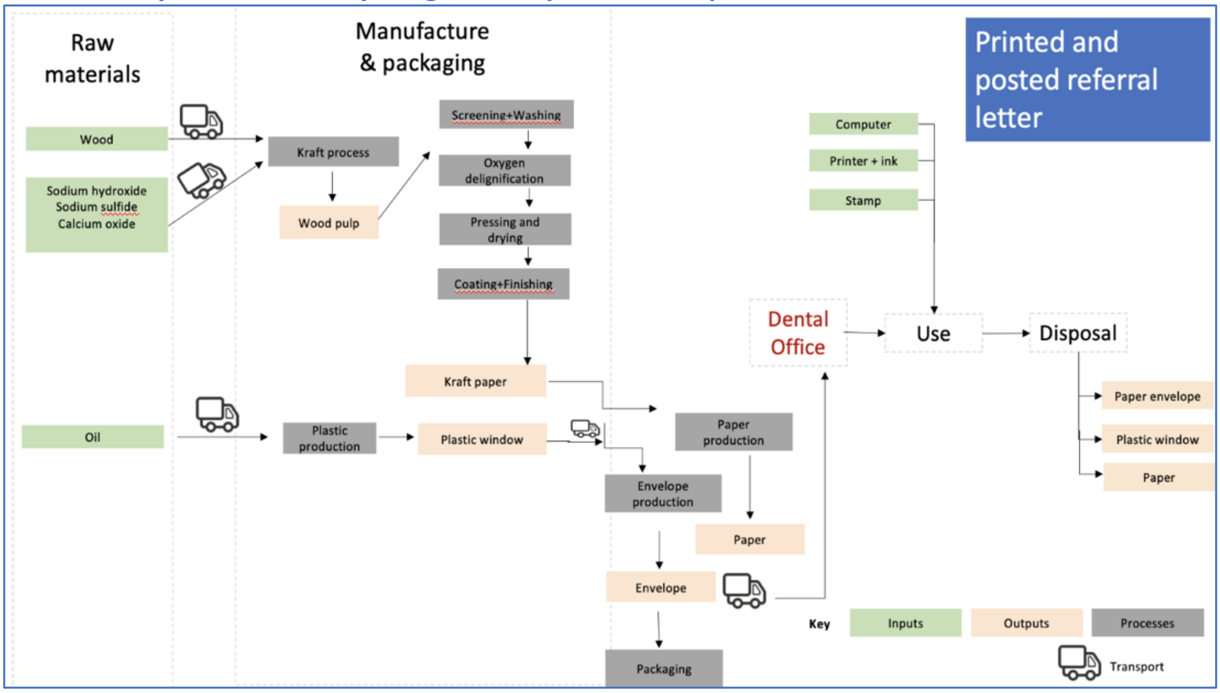

FIGURE 3 : System Boundary Diagram for paper for scrap work

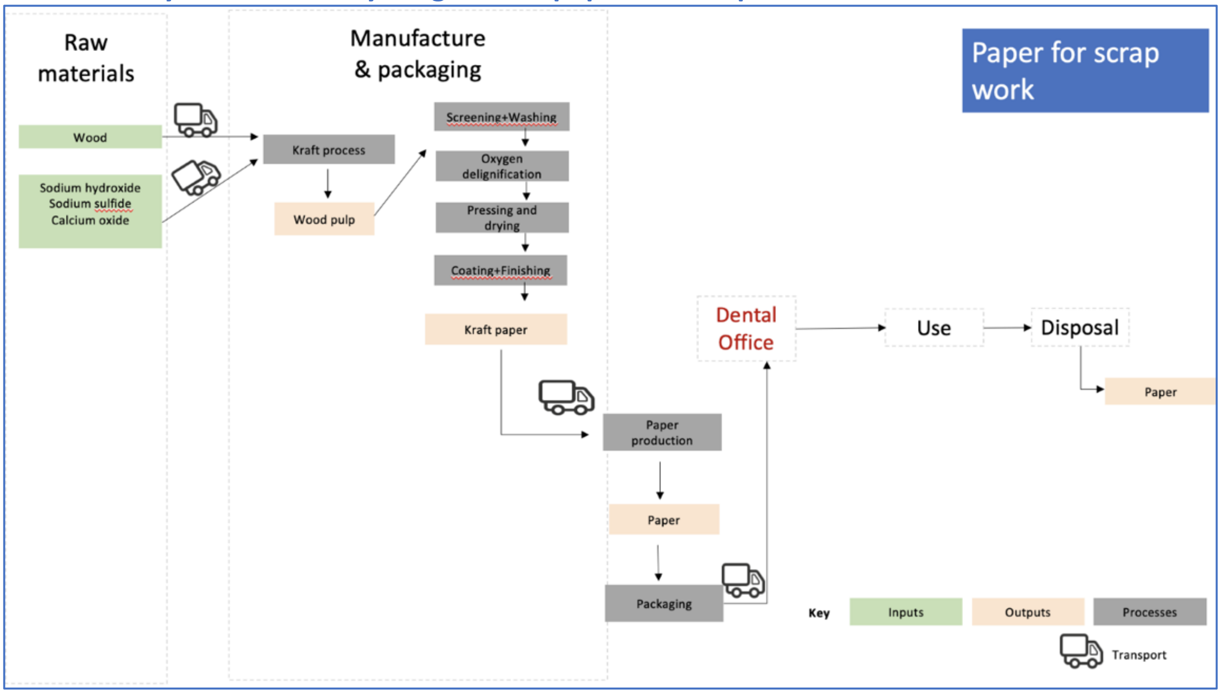

FIGURE 4: System Boundary Diagram for paper for reusing paper envelopes

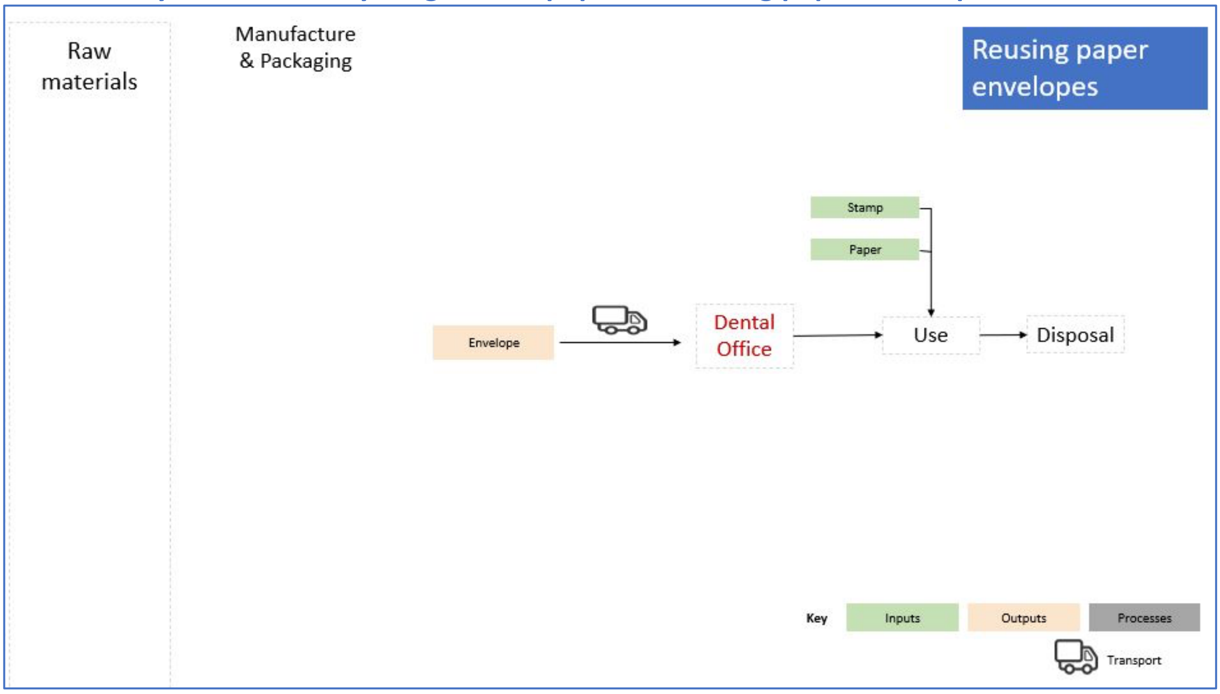

FIGURE 5 : System Boundary Diagram for buying new paper envelopes

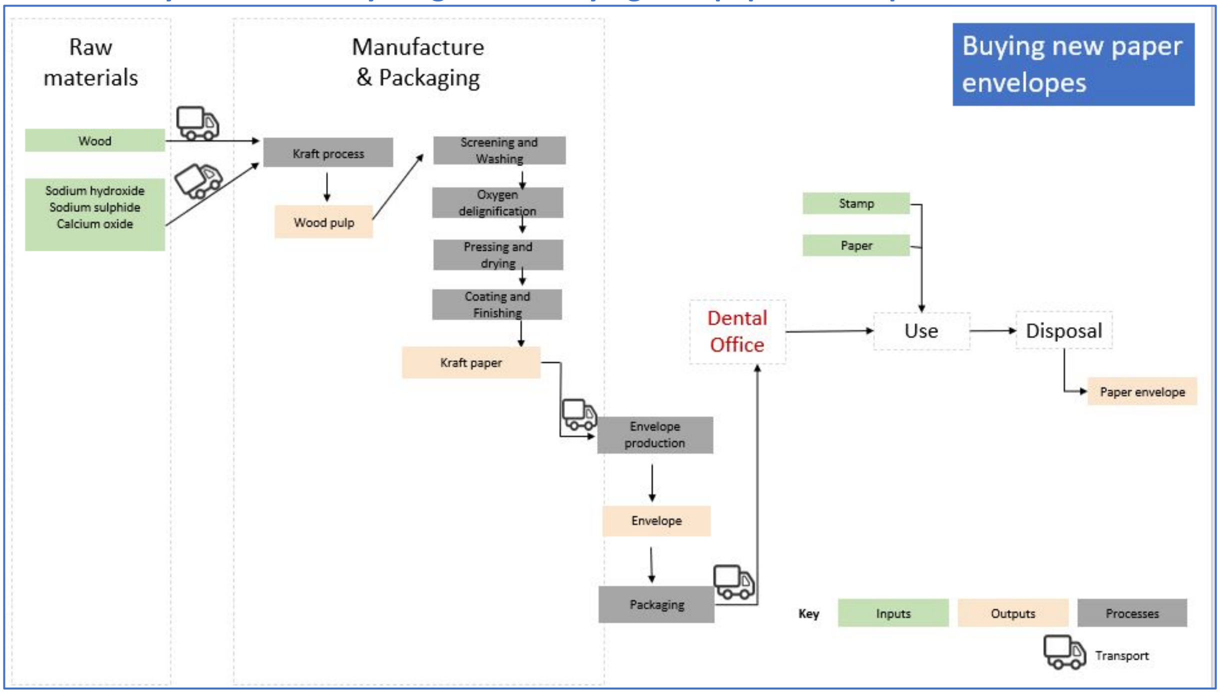

FIGURE 6: System Boundary Diagram for printing/photocopying on paper

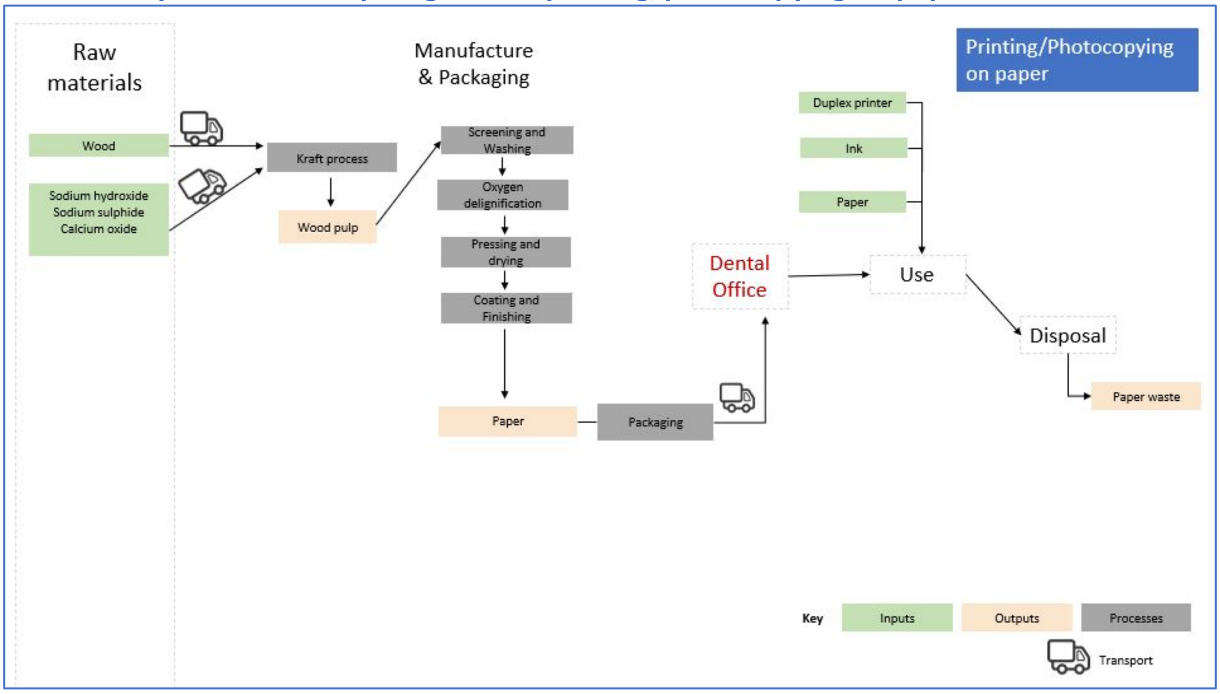

FIGURE 7: System Boundary Diagram for paper envelope with no plastic window

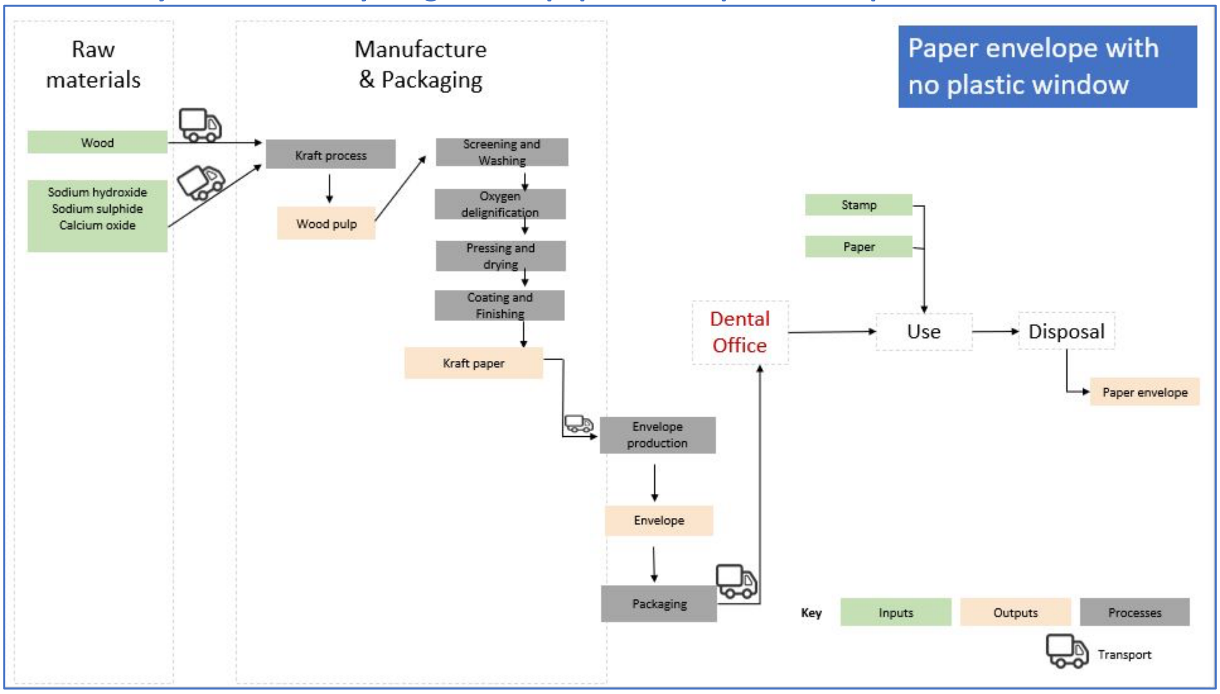

FIGURE 8: System Boundary Diagram for paper envelope with plastic window

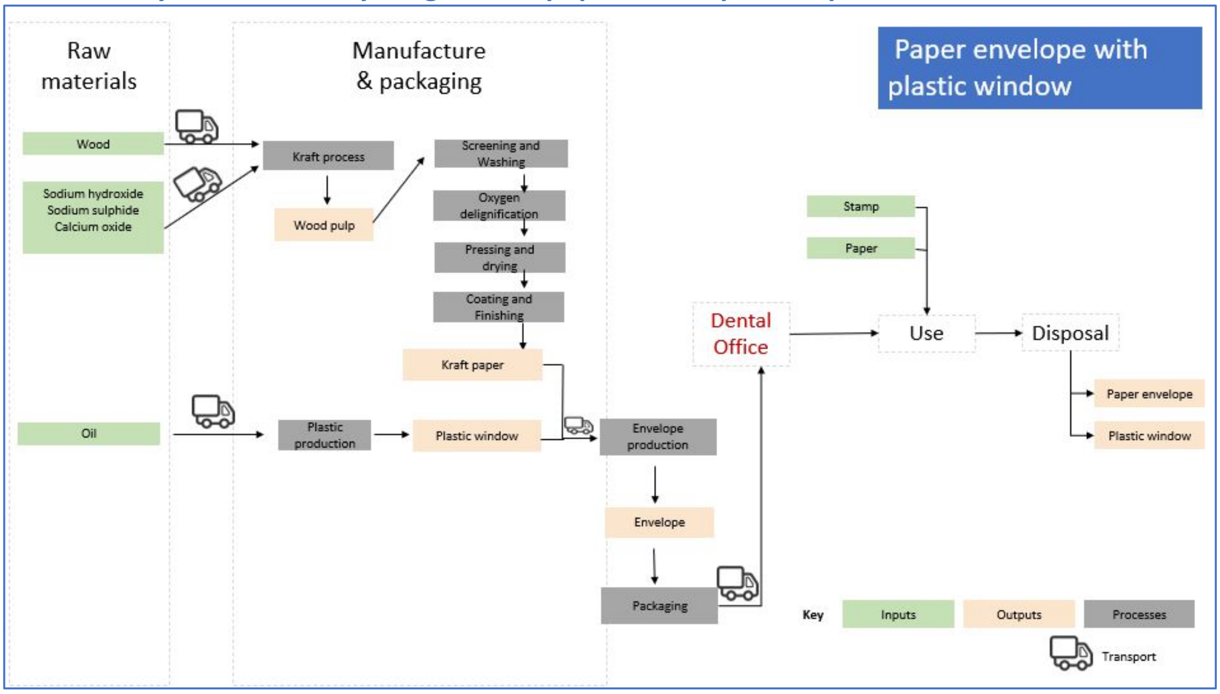

FIGURE 9: System Boundary Diagram for regular flush toilet

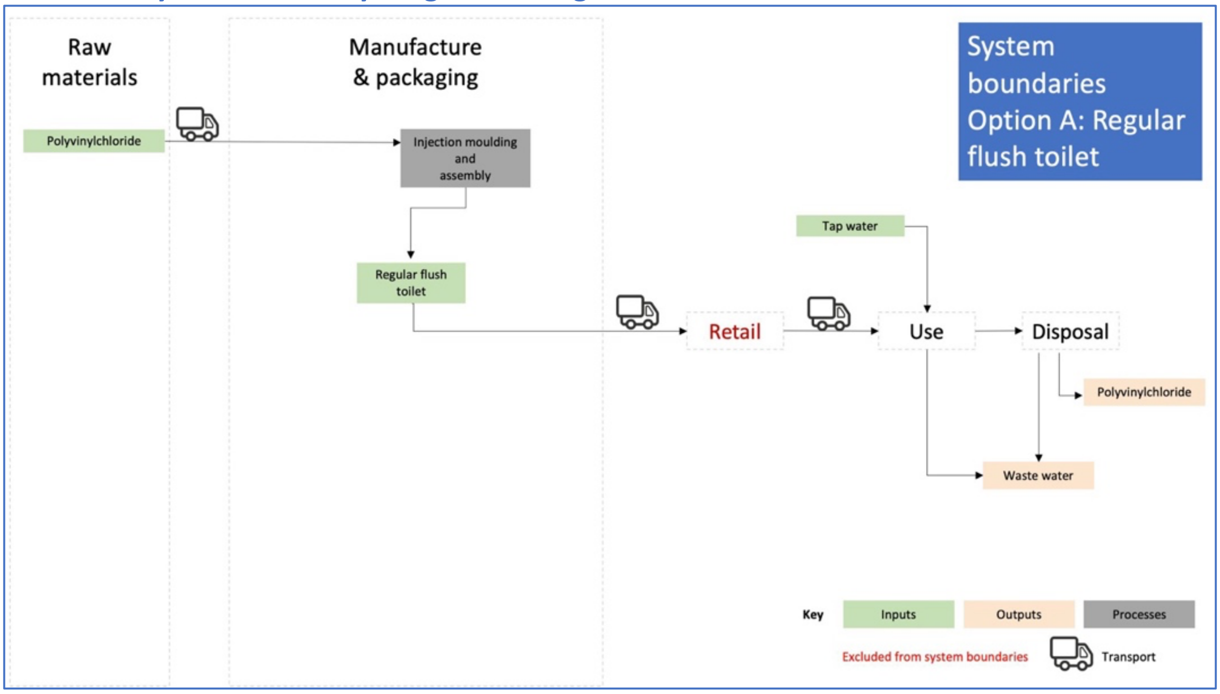

FIGURE 10: System Boundary Diagram for dual flush toilet

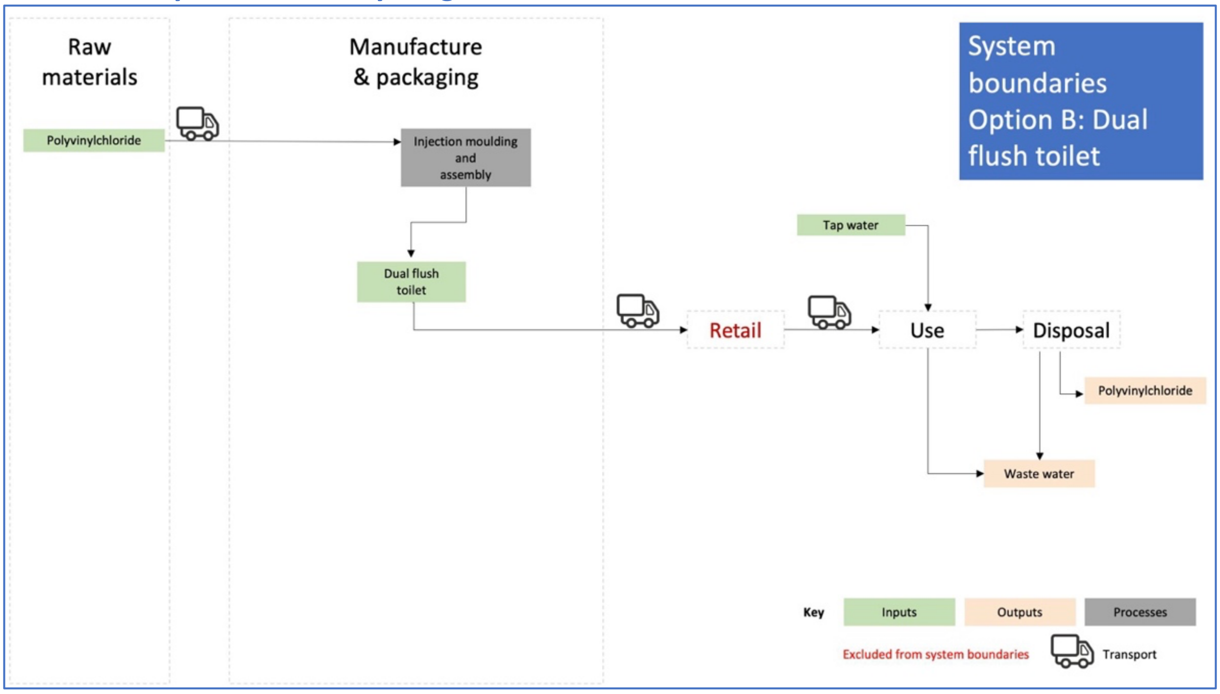

FIGURE 11 : System Boundary Diagram for rain collecting system

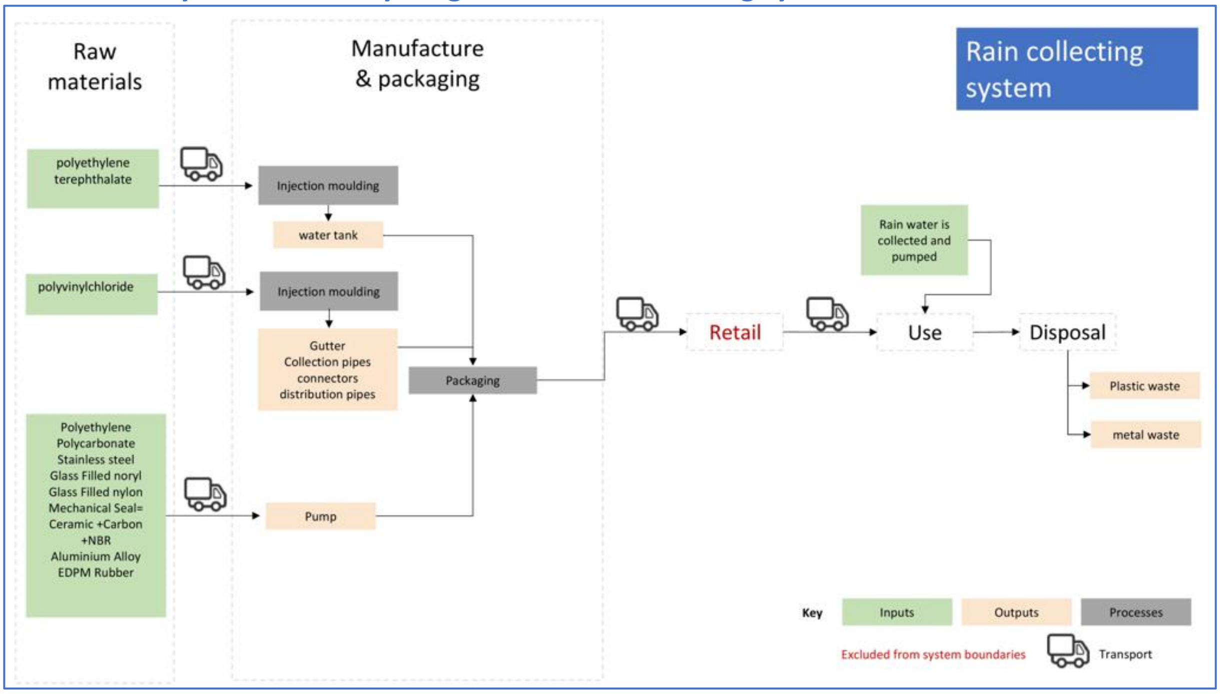

FIGURE 12: System Boundary Diagram for autoclaving, shredding and recycling toothbrush

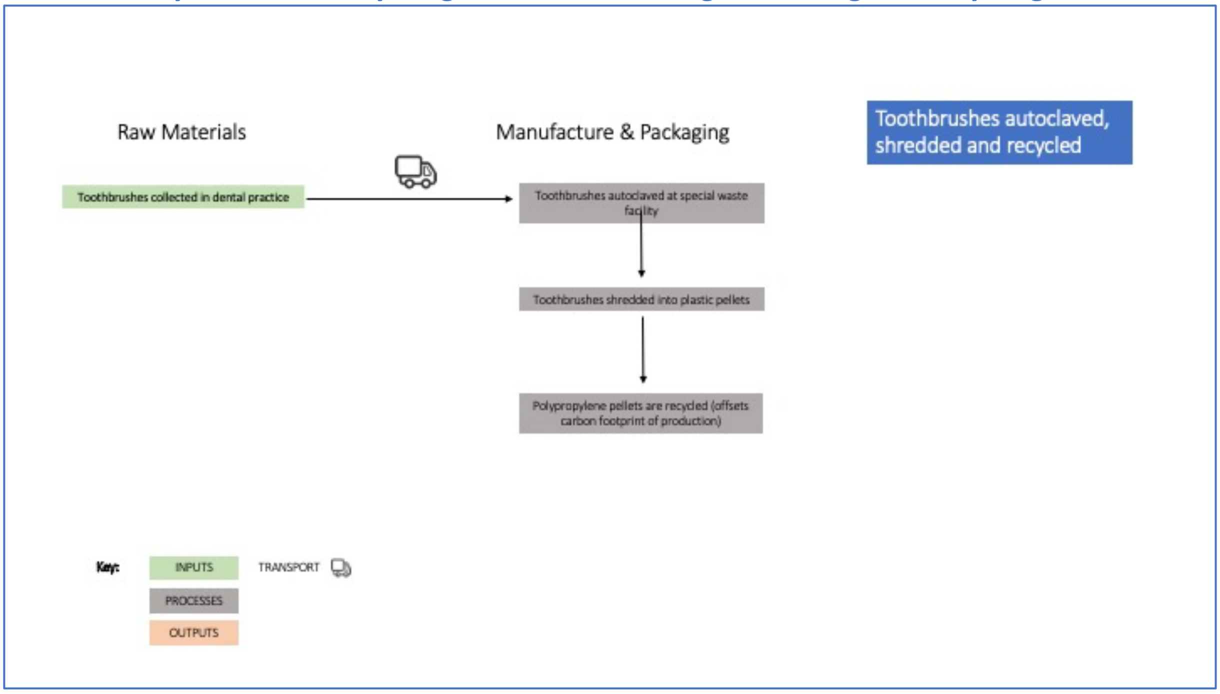

FIGURE 13: System Boundary Diagram for incinerating toothbrush

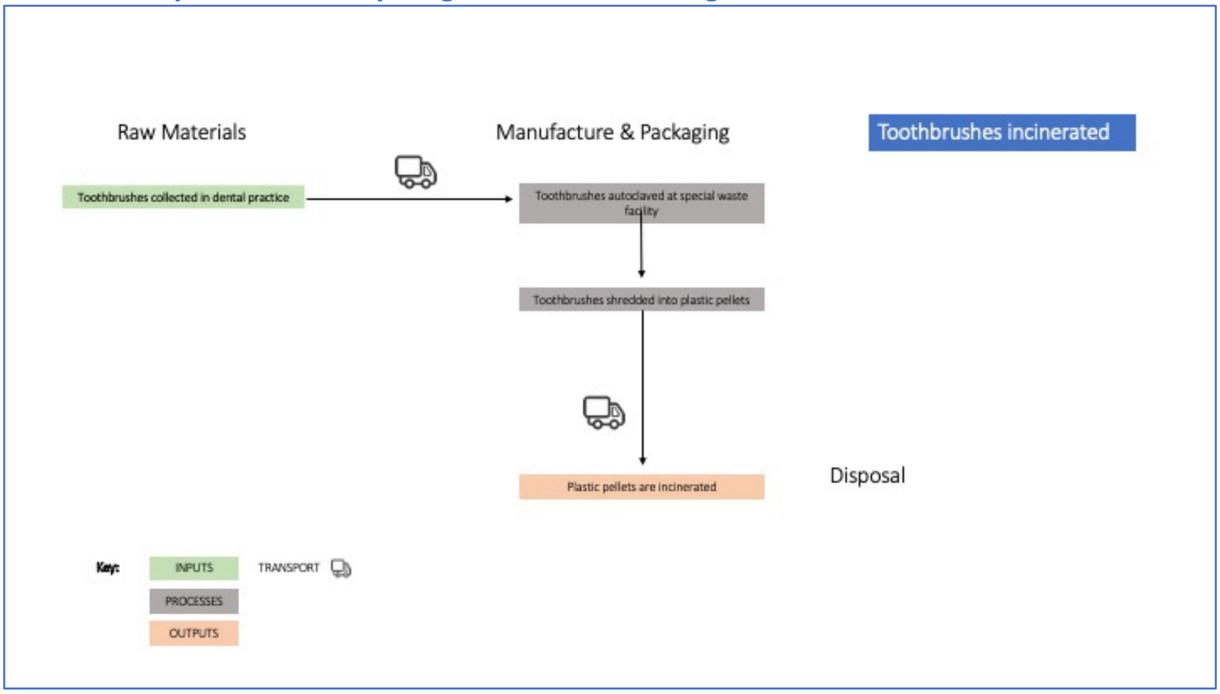

FIGURE 14 : System Boundary Diagram for disposable 3in1 syringe tip

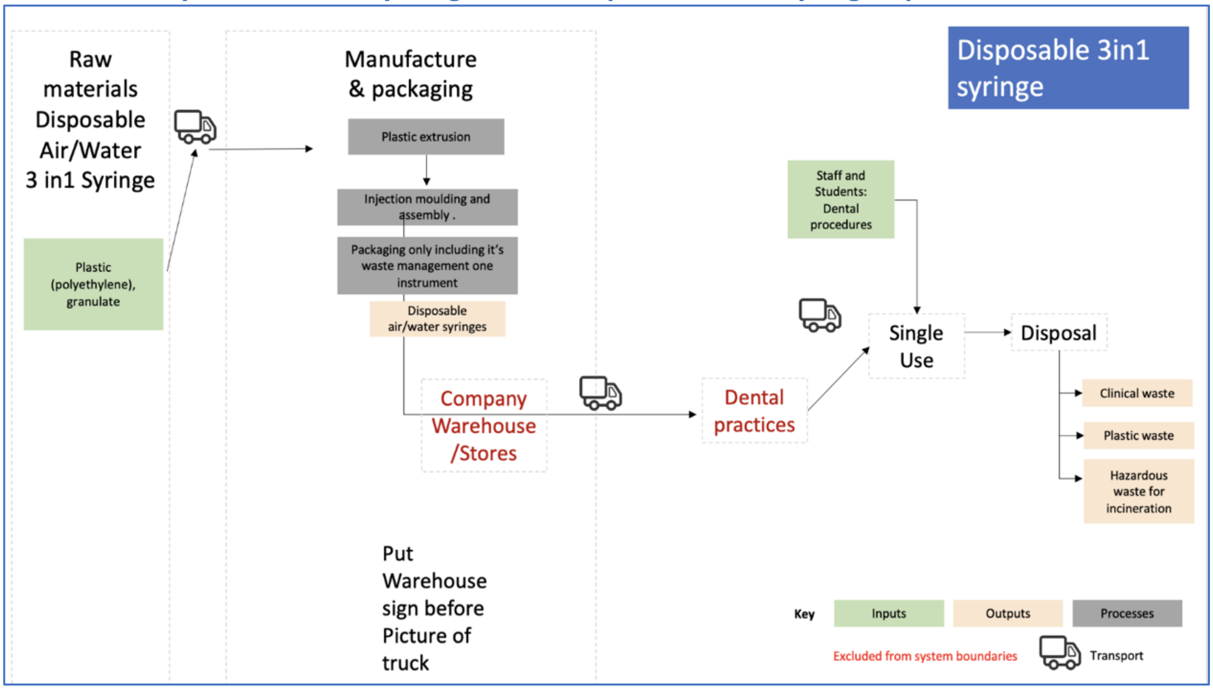

FIGURE 15 : System Boundary Diagram for metal 3in1 syringe tip

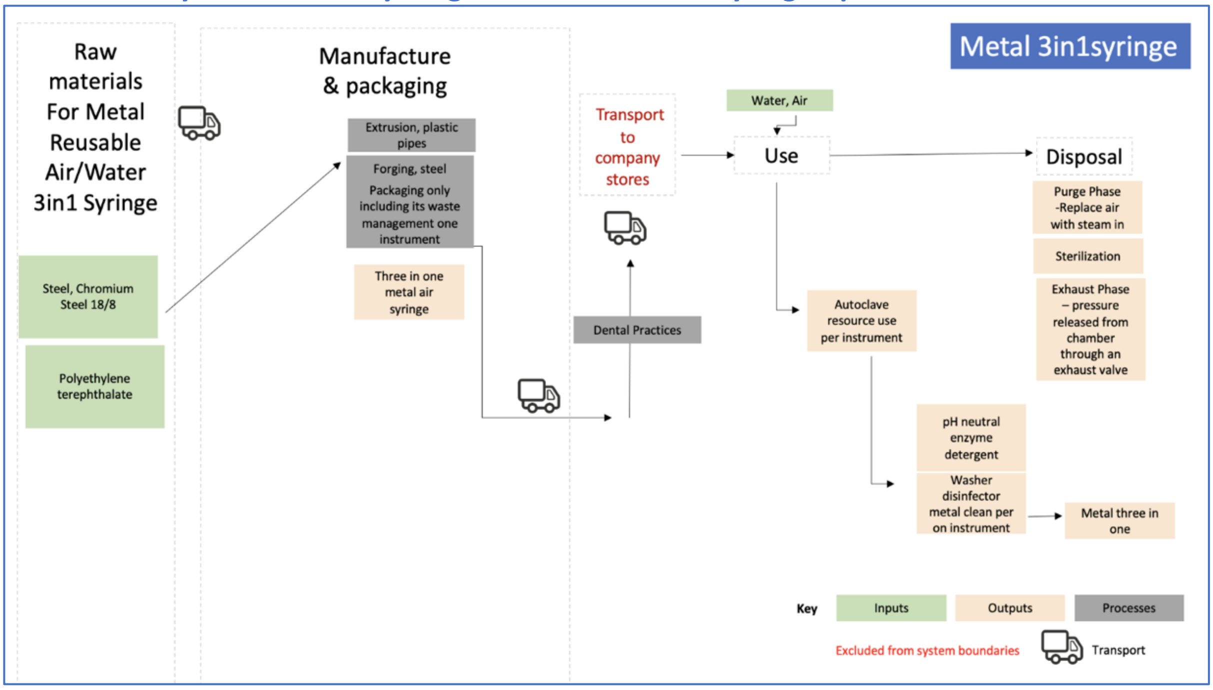

FIGURE 16: System Boundary Diagram for washing dishes with running water

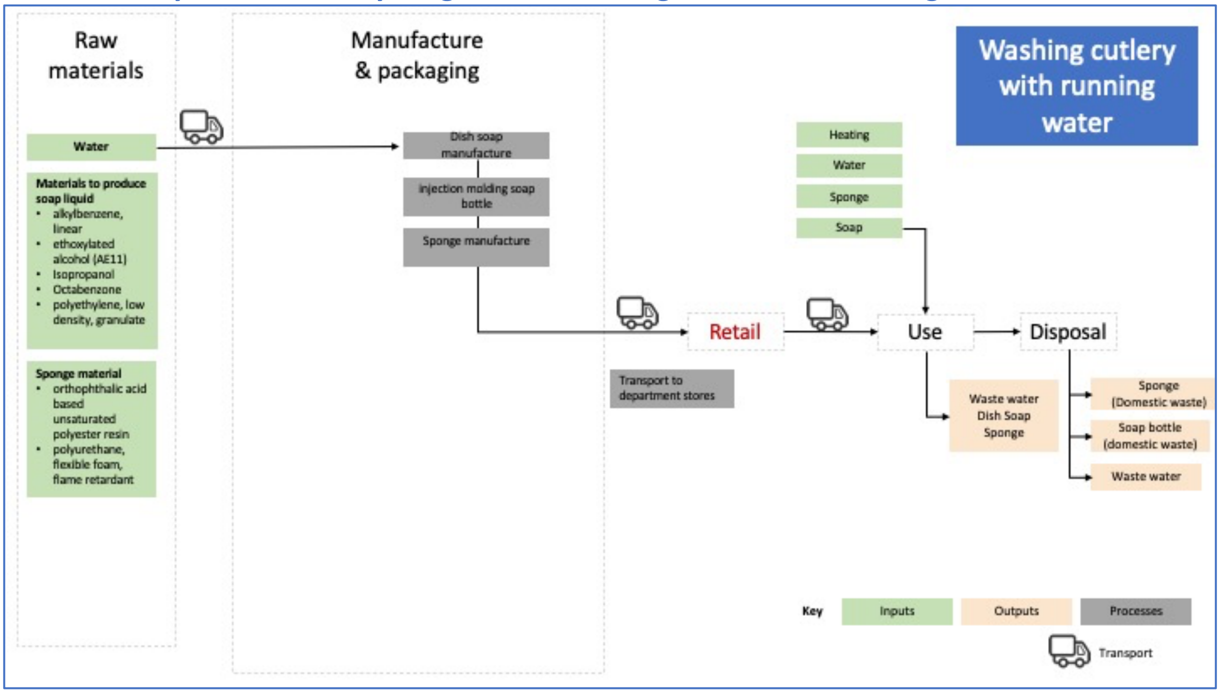

FIGURE 17: System Boundary Diagram for washing dishes in a dishwasher

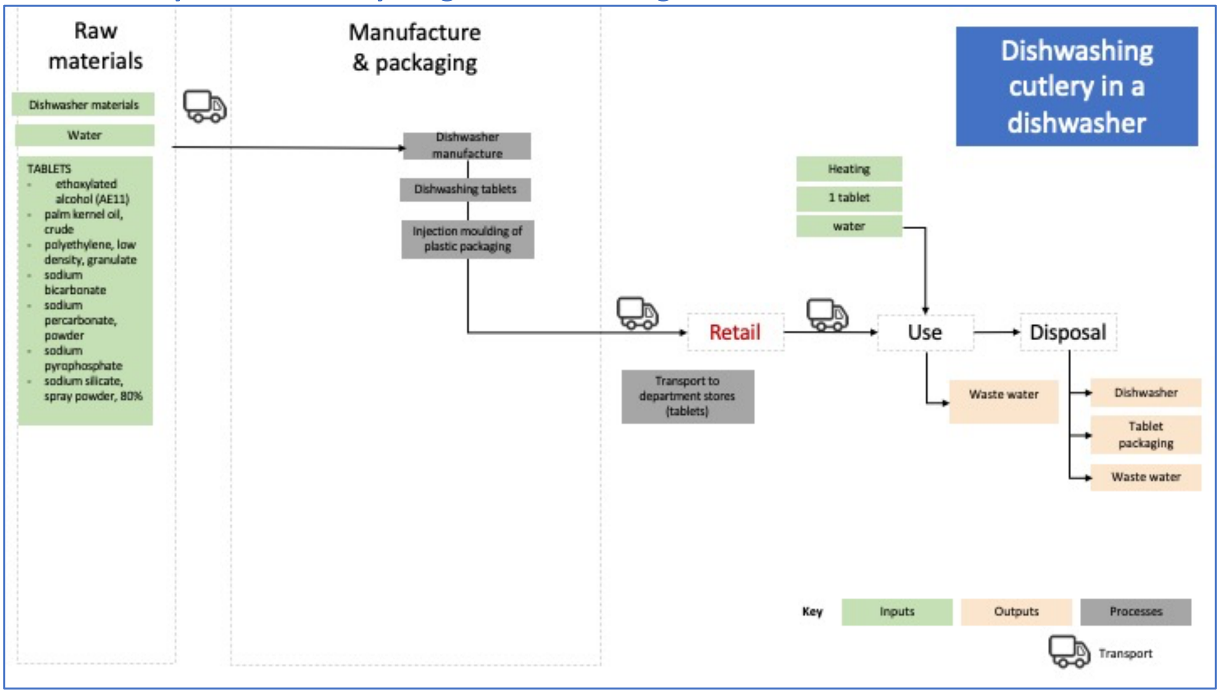

FIGURE 18 : System Boundary Diagram for washing dishes in a plugged sink

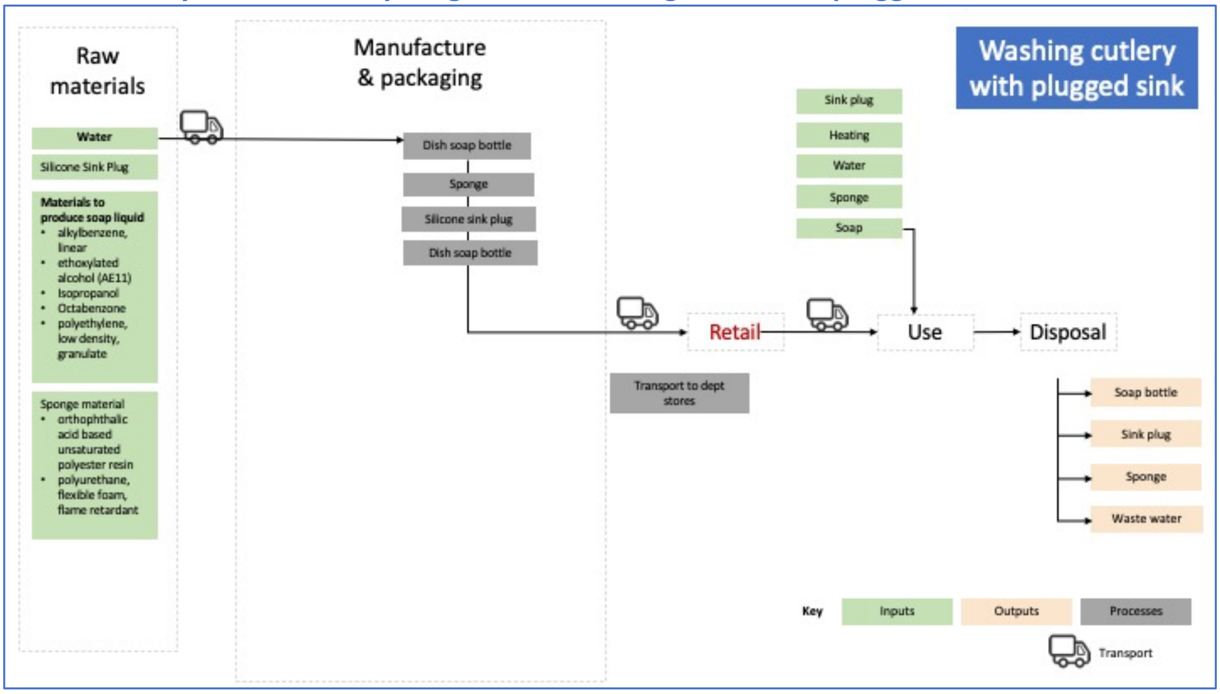

Supplementary Appendix 11

Contribution Analysis tables from the result of the LCA.

Functional unit one: E-mailing correspondence from the practice associated with one patient

Table 1: Breakdown Climate change: E-mailing appointment notice

E-mailing appointment notice	KgCO2eq
Computer operation	0.00083

Table 2: Breakdown Climate change: Posting appointment notice

Posting appointment notice	KgCO2eq
Paper and paper envelope	0.0052
Printer	0.00321
Computer operation	8.30E-04
Plastic window on envelope	0.00047
Disposal of paper	0.00036
Disposal of plastic window on envelope	0.00035
Transport	0.00021

Table 3: Breakdown Climate change: E-mailing referral letter

E-mailing referral letter	KgCO2eq
Computer operation	0.00414

Table 4: Breakdown Climate change: Posting referral letter

Posting referral letter	KgCO2eq
Computer operation	0.01243
Paper and paper envelope	0.0052
Printer	3.21E-03
Plastic window on envelope	0.00047
Disposal of paper	0.00036
Disposal of plastic window on envelope	0.00035
Transport	0.00021

Functional unit two: Paper use associated with one patient

Table 5: Breakdown Climate change: Keeping scrap paper

Keeping scrap paper	KgCO2eq
Paper	0.0028

Table 6: Breakdown Climate change: Disposing of scrap paper

Disposing of scrap paper	KgCO2eq
Paper	0.0028
Disposal of paper	0.0002

Table 7: Breakdown Climate change: Shredding confidential documents

Shredding confidential documents	KgCO2eq
Paper	0.0028
Disposal of paper	0.0002
Shredding	1.50E-04
Sign indicating items suitable for shredding	2.46E-05

Table 8: Breakdown Climate change: Shredding all documents

Shredding all documents	KgCO2eq
Paper	0.0056
Disposal of paper	0.00039
Shredding	3.00E-04

Table 9: Breakdown climate change: Printing/Photocopying one side of paper

Printing/Photocopying one side of paper	KgCO2eq
Paper	0.0056
Printer	0.00321
Computer	8.30E-04
Disposal of paper	0.00039

Table 10: Breakdown Climate change: Printing/Photocopying both sides of paper

Printing/Photocopying both sides of paper	KgCO2eq
Paper	0.0028
Printer	0.00321
Computer	8.30E-04
Disposal of paper	0.0002

Functional unit three: Water consumed in toilets in a patient visit

Table 11: Breakdown climate change: dual flush valve

Dual flush	KgCO2eq
Wastewater treatment	0.00102
Tap water production	0.00077

Table 12: Breakdown climate change: regular flush valve

Regular flush	KgCO2eq
Wastewater treatment	0.00339
Tap water production	0.00154

Functional unit four: Water used in the practice during a single patient visit

Table 13: Breakdown climate change: Tap water usage

Tap water usage	KgCO2eq
treatment of wastewater	0.02081
Tap water production	0.01251

Table 14: Breakdown climate change: Rainwater collection system

Waste from rain collecting system	KgCO2eq
Waste of pump	0.00172
Rain collecting system	0.00159

Functional unit five: Managing the waste from a toothbrush from a patient

Table 15: Breakdown climate change: Toothbrush waste autoclaving, shredding and recycling

Toothbrush waste autoclaving, shredding and recycling	KgCO2eq
Shredding waste	0.00087
Autoclaving of waste	0.00086
Transport from use to waste plant (40 miles)	0.00012

Table 16: Breakdown climate change: Toothbrush waste incineration

Toothbrush waste incineration	KgCO2eq
Treatment of waste - incineration	0.04566
Shredding waste	0.00087
Autoclaving of waste	0.00086
Transport from use to waste plant (40 miles)	0.00024

Functional unit six: Air-water syringes used in a single patient visit

Table 17: Breakdown climate change: Disposable air water syringe tip

Disposable air water syringe tip	KgCO2eq
Incineration, treatment for hazardous waste	0.016
Polypropylene	0.01569
Transport for one instrument per gram	0.0125
Injection moulding	0.00959
Extrusion	0.00283
Packaging	0.00146

Table 18: Breakdown climate change: metal air water syringe tip

Metal 3 in 1 air water syringe tip	KgCO2eq
Autoclave	0.01297
Washer disinfector	0.00582
Steel	0.00010
Incineration of hazardous waste	5.80522E-5
Transport for one instrument	4.35332E-5
Forging steel	2.54193E-5
Polyethylene	6.95917E-5
Packaging	4.74370E-6
Extrusion	0.00099

Functional unit seven: Water consumed in washing dishes in staff canteen per patient visit

Table 19: Breakdown climate change: Dishwashing cutlery in dishwasher

Dishwashing cutlery in dishwasher	KgCO2eq
Dishwashing tablet	5.08E-02
Dishwasher	2.18E-02
Electricity	2.98E-03
Mains water	7.10E-04
Waste water	4.30E-04

Table 20: Breakdown climate change: Washing cutlery in running water

Washing cutlery in running water	KgCO2eq
Heating	0.00689
Soap	0.00133
Waste water	9.70E-04
Mains water	0.00058
Sponges Production	1.70E-04
Waste sponge	1.09E-04

Table 21: Breakdown climate change: Washing cutlery in plugged sink

Washing cutlery in plugged sink	KgCO2eq
Solid waste	0.0215
Heating	0.00937
Soap	1.33E-03
Waste water	0.00132
Waste plastic	0.00123
Mains water	0.00079
Sponge	1.70E-04
Silicone sink plug	3.70E-06
